# Retention of OsNMD3 in the cytoplasm disturbs protein synthesis efficiency and affects plant development in rice

**DOI:** 10.1093/jxb/eru150

**Published:** 2014-04-10

**Authors:** Yanyun Shi, Xiangling Liu, Rui Li, Yaping Gao, Zuopeng Xu, Baocai Zhang, Yihua Zhou

**Affiliations:** State Key Laboratory of Plant Genomics, Institute of Genetics and Developmental Biology, Chinese Academy of Sciences, Beijing 100101, China

**Keywords:** Agronomic trait, dominant negative, OsNMD3, ribosome biogenesis, rice, translational efficiency.

## Abstract

The ribosome is the basic machinery for translation, and biogenesis of ribosomes involves many coordinated events. However, knowledge about ribosomal dynamics in higher plants is very limited. This study chose a highly conserved *trans*-factor, the 60S ribosomal subunit nuclear export adaptor NMD3, to characterize the mechanism of ribosome biogenesis in the monocot plant *Oryza sativa* (rice). *O. sativa* NMD3 (OsNMD3) shares all the common motifs and shuttles between the nucleus and cytoplasm via CRM1/XPO1. A dominant negative form of OsNMD3 with a truncated nuclear localization sequence (OsNMD3^ΔNLS^) was retained in the cytoplasm, consequently interfering with the release of OsNMD3 from pre-60S particles and disturbing the assembly of ribosome subunits. Analyses of the transactivation activity and cellulose biosynthesis level revealed low protein synthesis efficiency in the transgenic plants compared with the wild-type plants. Pharmaceutical treatments demonstrated structural alterations in ribosomes in the transgenic plants. Moreover, global expression profiles of the wild-type and transgenic plants were investigated using the Illumina RNA sequencing approach. These expression profiles suggested that overexpression of OsNMD3^ΔNLS^ affected ribosome biogenesis and certain basic pathways, leading to pleiotropic abnormalities in plant growth. Taken together, these results strongly suggest that OsNMD3 is important for ribosome assembly and the maintenance of normal protein synthesis efficiency.

## Summary

Trapping OsNMD3 in the cytoplasm by overexpression of the truncated nuclear localization sequence form in rice alters ribosome assembly and protein synthesis efficiency, leading to pleiotropic abnormalities in plant growth.

## Introduction

The ribosome, an apparatus for translation, is universally composed of one large and one small subunit. In bacteria, the large (50S) and small (30S) subunits contain three rRNAs (23S and 5S in the 50S subunit and 16S in the 30S subunit) and tens of ribosomal proteins (RPs), whereas eukaryotic large (60S) and small (40S) subunits are assembled by rRNAs of different sizes (25S, 5.8S, and 5S for the 60S subunit and 18S rRNA for the 40S subunit) and a different number of RPs (Trapman *et al.*, 1975; Shajani *et al.*, 2011). Both subunits have distinct functions: the small subunit is responsible for decoding genetic information, whereas the large subunit is required for polypeptide synthesis (Steitz, 2008; Schmeing and Ramakrishnan, 2009). As the basic machinery for protein synthesis, increasing evidence has demonstrated that ribosome biogenesis is pivotal for human health and organism growth. Mutations in any essential components interfere with protein synthesis and cause genetic diseases in humans and growth abnormalities in plants.

However, the process of ribosome biogenesis is very complicated and highly coordinated. Although studies on prokaryotic and yeast ribosome structure and function over the past 50 years have provided insights into the mechanisms of ribosome biogenesis, knowledge about ribosome dynamics remains rudimentary. It is known that ribosome biogenesis begins with the transcription of pre-rRNA and the assembly of pre-ribosome particles in the nucleolus followed by transport from the nucleus and maturation in the nucleoplasm and/or cytoplasm (Kressler *et al.*, 2010; Panse and Johnson, 2010). In addition to pre-rRNA processing, folding, and interaction with ribosomal proteins, eukaryotic ribosome biogenesis requires the involvement of more than 200 nonribosomal factors. The functions of these *trans*-acting factors include modification, intracellular transport, and energy production (Kressler *et al.*, 2010; Panse and Johnson, 2010), and some of these roles are highly conserved in eukaryotic organisms.

Nuclear export of the 60S subunit is a relatively well-studied process related to ribosome biogenesis in yeast and animal cells. The *trans*-acting factors XPOl/CRM1, the GTPase Ran, and export adaptors are required for this process (Thomas and Kutay, 2003). In eukaryotic cells, three different types of nuclear export adaptors have been reported: NMD3, which acts as a bridge between CRM1 and the 60S particle (Ho *et al.*, 2000; Hedges *et al.*, 2006), Mex67-Mtr2, which binds to nucleoporins and 5S rRNA (Strässer *et al.*, 2000; Yao *et al.*, 2007), and Arx1, which interacts with RPL25 (Hung and Johnson, 2006; Bradatsch *et al.*, 2007). Among the three factors, NMD3 is highly conserved across yeast, animals, and plants, not only with respect to protein structure but also with respect to the function. NMD3 was first identified as a component of the nonsense-mediated mRNA decay (NMD) pathway (He and Jacobson, 1995) and was further demonstrated to be involved in 60S pre-ribosome export and maturation (Belk *et al.*, 1999; Ho and Johnson, 1999; Sengupta *et al.*, 2010). Due to the presence of a nuclear localization sequence (NLS) and a nuclear export sequence (NES) at the C terminus, NMD3 can shuttle in and out of the nucleus (Ho *et al.*, 2000; Gadal *et al.*, 2001). The leucine-rich NES was further found to interact with CRM1 and mediate the export of the 60S subunit from the nucleus (Gadal *et al.*, 2001; Hedges *et al.*, 2006). Deletion of the C-terminal NES from NMD3 resulted in accumulation of RPL25 in the nucleus (Ho *et al.*, 2000). The N-terminal NMD3 is also highly conserved and contains four Cys–*X*2–Cys repeats, which are most likely essential for its interaction with rRNA and/or RPs of 60S subunits (Teakle and Gilmartin, 1998). Following nuclear export, the pre-60S particles mature in the cytoplasm via removal of NMD3 through interaction with RPL10 and the GTPase Lsg1. NMD3 is one of the factors that blocks the interface of the 60S subunit to prevent it from binding to 40S subunits (Gartmann *et al.*, 2010; Sengupta *et al.*, 2010). Removal of NMD3 is thus an important cytoplasmic maturation step of 60S subunits and represents a critical checkpoint for quality control of nascent ribosomes and ensuring translational competence (Karbstein, 2013). Recently, an increasing number of studies have focused on cytoplasmic maturation events due to their importance and complexity (Panse and Johnson, 2010). Disruption of the components involved in cytoplasmic maturation will lead to the inability to assemble functional ribosomes, leading to a human health crisis (Burwick *et al.*, 2011; Vlachos *et al.*, 2012).

The current knowledge of ribosome biogenesis is mainly based on studies in yeast and animals (Byrne, 2009), although increasing evidence has shown that ribosomal proteins play essential roles in plant growth (Degenhardt and Bonham-Smith, 2008; Pinon *et al.*, 2008; Horiguchi *et al.*, 2011; Szakonyi and Byrne, 2011). The NMD3 protein was found to be evolutionarily conserved in yeasts, animals, and plants, and it has been annotated in many plant species. As a conserved *trans*-acting factor, NMD3 may provide a breakthrough point for the investigation of ribosome biogenesis in plants, of which the relevant knowledge is very limited. A pioneering work conducted on *Arabidopsis* NMD3 revealed that this protein is involved in CRM1-mediated nuclear export of the 60S subunit; however, the detailed mechanism of this process may be different from that in yeast and animals (Chen *et al.*, 2012). Overexpression of a NES-deleted form of AtNMD3 induced the accumulation of RPL28A-YFP in the nucleus and caused a defect in secondary-cell-wall thickening. This study demonstrated the function of AtNMD3 in pre-60S nuclear export. However, the role of AtNMD3 in pre-60S maturation remains elusive. Rice is an important crop and a model organism of the Gramineae, and it possesses one conserved NMD3 sequence. It will be interesting to investigate whether this protein performs a related and/or distinct function from NMD3 proteins reported in other species.

This work characterized the identity and function of *Oryza sativa* NMD3 (OsNMD3) by transient expression of its wild-type, NES-deleted, and NLS-deleted forms in rice protoplasts and by generation of transgenic plants harbouring the related transgenes. Overexpression of the NLS-truncated form of OsNMD3 trapped the resulting protein in the cytosol and interfered with pre-60S ribosome maturation and translational efficiency. Through RNA sequencing and phenotype characterization, changes in gene expression and important agronomic traits have been analysed. These studies enhance the understanding of the mechanisms of plant ribosomal biogenesis and provide the opportunity for improving agronomic traits via manipulation of protein synthesis.

## Materials and methods

### Plant materials and growth conditions

For generation of the transgenic plants, the full length CDS of wild-type *OsNMD3* as well as the NLS-truncated form were amplified by PCR using the primers (Supplementary Table S1 available at *JXB* online). After sequencing confirmation, the fragments were in-frame fused with GFP at the C-terminus and inserted between the CaMV 35S promoter and the nopaline synthase (NOS) terminator in the *pCAMBIA* 1300 vector. The resulting constructs were transfected into *Agrobacterium tumefaciens* EHA105 and introduced into the wild-type variety Nipponbare. The transgenic plants were cultivated in experimental fields of the Institute of Genetics and Developmental Biology in Beijing or Sanya (Hainan Province, China) during natural growing seasons.

### Bioinformatics analysis

Annotation of OsNMD3 was performed using the Rice Genome Annotation Project (http://rice.plantbiology.msu.edu/). Pfam (www.sanger.ac.uk) and SMART (http://smart.embl-heidelberg.de) searching were used to predict the motifs of OsNMD3. An unrooted phylogenetic tree of NMD3 homologues was generated using MEGA 5 with 1000 bootstrap replicates (Tamura *et al.*, 2011). Bootstrap values are presented as percentages. Multiple alignments were constructed with the ClustalX and Jalview programs (Waterhouse *et al.*, 2009).

### Rice protoplast transformation

GFP was fused to the C-terminus of wild-type OsNMD3 as well as the NLS-truncated and NES-truncated forms. The genes were inserted between the CaMV 35S promoter and the NOS terminator in the pUC18 vector. The generated constructs were transformed into rice protoplasts, which were isolated from wild-type plants as previously described (Zhou *et al.*, 2009). A vector harbouring GFP alone was transformed into the same protoplasts, serving as a negative control. Nuclei were stained with 4′,6-diamidino-2-phenylindole (DAPI, 1 μg ml^–1^, Sigma). The fluorescent signals were observed with a confocal laser scanning microscope (Leica TCS SP5).

For the transactivation assay, protoplasts were prepared from 14-d-old wild-type and transgenic L1 seedlings as described above. Reporter plasmid DNA (6 μg, containing a 5× upstream activating sequence and a 35S promoter driving firefly luciferase) and effector plasmid DNA (containing a 35S promoter driving the GAL4 DNA-binding domain fused VP16) (Ohta *et al.*, 2000) were cotransformed by the PEG method (Zhou *et al.*, 2009). The transformation efficiency was normalized by introducing 0.1 μg pPTRL vector plasmid DNA (containing a 35S promoter driving a *Renilla* luciferase gene) as an internal control. The transformed protoplasts were incubated at 28 °C for 18h. The firefly luciferase activity was detected using the GloMax 20/20 Luminometer according to the operation manual provided with the dual-luciferase reporter assay system (Promega). Data were presented as mean of three biological replicates.

### Ribosome profile analysis

Isolation of plant ribosomes by sucrose density-gradient centrifugation was carried out as described previously (Mustroph *et al.*, 2009) with some minor modifications. Ten-d-old seedlings (6g) were ground in liquid nitrogen and homogenized in 25ml ribosome extraction buffer (0.2M Tris pH 9.0, 0.2M KCl, 0.025M EGTA, 0.035M MgCl_2_, 1% Triton X-100, 1% Brij-35, 1% NP40, 1% Tween 20, 1% sodium deoxycholate, 1% polyoxyethylene 10 tridecyl ether, 5mM dithiothreitol (DTT), 50 μg ml^–1^ cycloheximide, 50 μg ml^–1^ chloramphenicol, 1mM phenylmethylsulphonyl fluoride, 0.5 μg ml^–1^ heparin). The samples were incubated on ice for 10min with occasional shaking. After filtering through four layers of sterile Miracloth, the mixture was centrifuged at 10 000 *g* (4 °C) for 30min. The supernatant (20ml) was overlaid on the top of sucrose cushion buffer (0.4M Tris pH 9.0, 0.2M KCl, 5mM EGTA, 35mM MgCl_2_, 1.75M sucrose, 5mM DTT, 50 μg ml^–1^ cycloheximide, 50 μg ml^–1^ chloramphenicol) and centrifuged at 116 000 *g* (4 °C) for approximately 18h. The pellet was resuspended in cold resuspension buffer (0.2M Tris pH 9.0, 0.2M KCl, 25mM EGTA, 35mM MgCl_2_, 5mM DTT, 50 μg ml^–1^ cycloheximide, 50 μg ml^–1^ chloramphenicol) and incubated on ice for 30min. After separation at 12 000 *g* (4 °C) for 2min, the supernatant (300 μl) was layered on a sucrose gradient (5–50% sucrose, 0.2M Tris (pH 8.4), 0.2M KCl, 0.1M MgCl_2_, 5 μg ml^–1^ cycloheximide, 5 μg ml^–1^ chloramphenicol), and centrifuged at 116 000 *g* (4 °C) for 150min using a Beckman SW41 Ti rotor. Gradient fractions were collected manually (BioComp Gradient Fractionator) from the top of the gradient. The optical density of each fraction was measured by UV absorbance at 260nm. Proteins in each fraction were precipitated in two volumes of 99% ethanol at 4 °C overnight. After washing once with 70% ethanol, the protein pellets were separated by 12% sodium dodecyl sulphate-polyacrylamide gel electrophoresis (SDS-PAGE) and probed with the primary antibodies anti-OsNMD3, anti-hsRPL4, anti-hsRPS14, and anti-GFP followed by a horseradish peroxidase-conjugated anti-rabbit IgG secondary antibody. The anti-hsRPL4 and anti-hsRPS14 were purchased from Proteintech and the anti-GFP was purchased from Roche.

### Western blotting

The nuclear proteins were extracted as previously described (Choudhary *et al.*, 2009). The protein samples were separated by SDS-PAGE, transferred onto nitrocellulose membranes, and analysed by protein blotting with anti-OsNMD3, anti-GFP, anti-Histone3, anti-RbcL, anti-hsRPL4, and anti-hsRPS14 antibodies, respectively. The anti-Histone3 and anti-RbcL were purchased from Agrisera. The secondary antibody, horseradish peroxidase-conjugated anti-rabbit IgG, was used for staining.

### Protein interaction assay

A luciferase complementation image assay was employed to investigate the interaction of OsNMD3 with RPLs (Chen *et al.*, 2008). The CDS of *OsNMD3*, *RPL10Ac1*, *RPL10B*, *RPL10C*, *RPL27Ab*, *RPL27Ac1*, and *RPL27Ac2* were amplified with the relevant primers (Supplementary Table S1) and inserted into pNLuc and pCLuc vectors. The sequences were verified, and the vectors were then introduced into *A. tumefaciens* EHA105. The leaves of 1-month-old *Nicotiana benthamiana* plants were infected with strains containing the constructs. After infection, the plants were maintained in the dark for 12h and subsequently incubated for additional 60h at a normal condition. The infected leaves were sprayed with 1mM luciferin and exposed under a low-light cooled CCD apparatus (NightOWL II LB983) for 10min. The images were harvested using IndiGO software.

### Pharmaceutical treatments

The germinated seeds of wild-type and transgenic plants were cultivated in liquid medium (l^–1^: 80mg NH_4_NO_3_, 93mg NaH_2_PO_4_·2H_2_O, 52.4mg K_2_SO_4_, 44.2mg CaCl_2_·2H_2_O, 122mg MgCl_2_·6H_2_O, 19mg FeEDTA, 3.01mg H_2_BO_3_, 2.17mg MnSO_4_· 5H_2_O, 0.075mg CuSO_4_·5H_2_O, 0.2008mg ZnSO_4_·7H_2_O, 0.024mg Na_2_MoO_4_) for 4 days. Then, the seedlings were cultivated in the liquid medium containing various concentrations of each antibiotic (0.8 μM cycloheximide, 1 μg ml^–1^ chloramphenicol, 50 μg ml^–1^ gentamicin, 100 μg ml^–1^ spectinomycin, 50 μg ml^–1^ spiramycin, 20 μg ml^–1^ tetracycline, 20 μM 5-fluorouracil) for 6 days. Cycloheximide and 5-fluorouracil were purchased from Sigma. The aerial parts of seedlings were cut and weighed. The relative freshweight was calculated by considering the freshweight without treatment as the reference. Data were presented as mean of 40 seedlings with standard errors.

### RNAseq analysis

Total RNA was extracted from the second internodes of development-matched wild-type and transgenic plants. The oligo(dT)-enriched mRNA was fragmented into segments approximately 200 nucleotides in length for cDNA synthesis. The library was then constructed and sequenced using an Illumina HiSeq 2000 instrument (BGI-Shenzhen). The raw sequence data were collected and filtered. Clean reads were obtained for the wild-type and L1 and L2 samples. After aligning to the rice reference cDNAs (MSU version 6.1) using SOAP aligner/soap2 (Li *et al.*, 2009), 6.35, 6.56, and 6.60 million reads from wild-type, L1 and L2 plants were evenly mapped. Level of gene expression was calculated using the RPKM (per kb per million reads) method. The significance of differentially expressed genes (DEGs) was determined as described previously (Audic and Claverie, 1997) using the criteria of false discovery rate <0.001 and absolute value of the log2 ratio >1. To perform pathway enrichment analysis using the Kyoto Encyclopaedia of Genes and Genomes (KEGG) database (http://www.genome.jp/kegg/), all 12342 DEGs were used to identify the significantly enriched metabolic or signal transduction pathways compared with the whole-genome background: 35 and 29 pathways were significantly enriched, with Q values <0.05. DEGs with more than 2-fold alterations are listed in Supplementary Data S1 and S2. The complete datasets are available under GEO accession number GSE55234 (http://www.ncbi.nlm.nih.gov/geo).

### Gene expression analysis

Total RNA was extracted from the second internodes of 2-month-old wild-type and transgenic plants using Plant RNA Purification Reagent (Invitrogen). Reverse transcription was conducted with 2 μg total RNA using a reverse transcriptional kit (Promega). Quantitative real-time PCR (qRT-PCR) was performed with a cycler apparatus (Bio-Rad) using FastStart Universal SYBR Green Master (Roche). The amplification protocol was 94 °C for 20 s and 40 cycles at 94 °C for 20 s, 58 °C for 20 s, and 72 °C for 20 s. The housekeeping gene *UBQ5* that has stable expression levels according to the RNAseq data was chosen as an internal control for normalization. Data were presented as mean of three replicates. The primer sequences are given in Supplementary Table S2 available at *JXB* online.

## Results

### A conserved NMD3 protein is found in rice

NMD3 is one of the highly conserved *trans*-acting factors and mediates the nuclear export of the 60S ribosomal subunit from yeast to plants (Ho and Johnson, 1999; Thomas and Kutay, 2003; Chen *et al.*, 2012). Through a BlastP search of the rice genome, the current work identified only one annotated NMD3 sequence with an open reading frame located at the locus LOC_Os10g42320 (Rice Genome Annotation Project, http://rice.plantbiology.msu.edu/) or Os10g0573900 (National Center for Biotechnology Information, http://www.ncbi.nlm.nih.gov/). An unrooted phylogenetic tree was further constructed including the NMD3 from 18 representative species of bacteria, animals, and plants using the neighbour-joining method (Supplementary Fig. S1A). OsNMD3 was clustered with sorghum NMD3 (SbNMD3) into a monophyletic clade that arose before the divergence of monocot and dicot phyla. Despite different evolutionary scenarios, OsNMD3 is conserved among the examined species. Within the length of 523 amino acids, OsNMD3 has four cysteine repeat motifs (Cx_2_C) at the N-terminus with an NLS (414–430 aa) and a leucine-rich NES (494–503 aa) at the C-terminus (Supplementary Fig. S1B). These motifs are highly conserved among all eukaryotic cells, indicating that OsNMD3 may play roles in 60S ribosome dynamics that are similar to those reported in other species.

### OsNMD3 is nucleocytoplasmically localized and shuttles via CRM1/XPO1

**Fig. 1.**
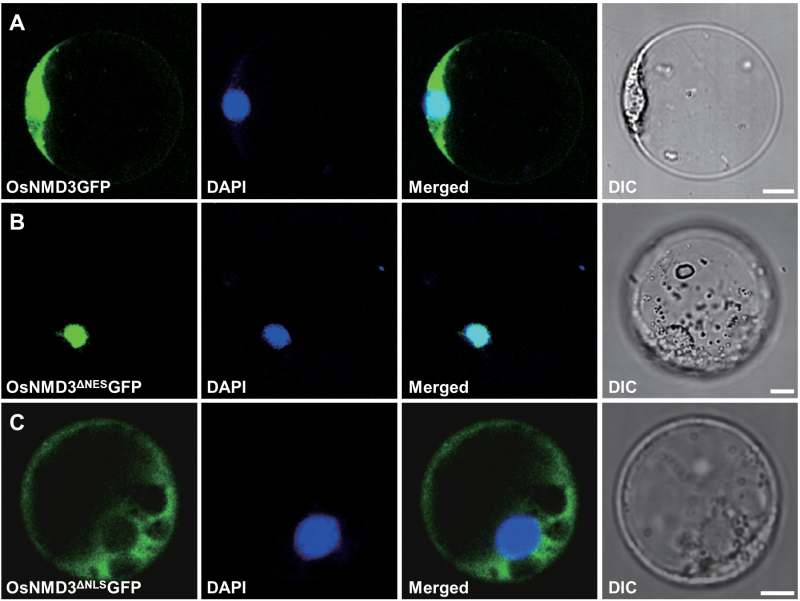
OsNMD3 is nucleocytoplasmically localized. (A–C) Rice protoplast cells expressing OsNMD3GFP (A), OsNMD3^ΔNES^GFP (B), and OsNMD3^ΔNLS^GFP (C). The cells are stained with DAPI to visualize the nucleus. GFP and DAPI signals are merged, and differential interference contrast (DIC) images are also shown. Bars, 5 µm.

The presence of NES and NLS motifs indicates that OsNMD3 is nucleocytoplasmically localized. This work therefore fused the green fluorescent protein (GFP) to the C-terminus of full-length OsNMD3 (OsNMD3GFP) and to the C-terminus of truncated OsNMD3 lacking either the NES (OsNMD3^ΔNES^GFP) or NLS (OsNMD3^ΔNLS^GFP) and transiently expressed the resulting constructs in rice protoplasts. Fluorescent signals of OsNMD3GFP were observed in both the nucleus and cytoplasm (Fig. 1A), whereas those of OsNMD3^ΔNES^GFP and OsNMD3^ΔNLS^GFP were trapped in the nucleus and cytoplasm, respectively (Fig. 1B, C).

**Fig. 2.**
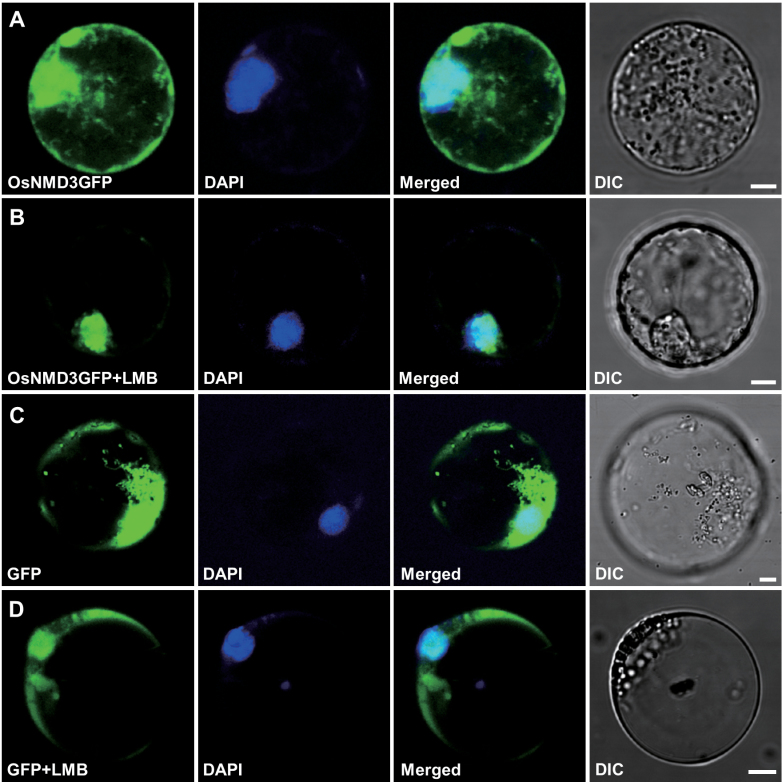
The effects of leptomycin B (LMB) on OsNMD3 distribution. (A and B) Rice protoplast cells expressing OsNMD3GFP were untreated (A) or treated (B) with LMB (+LMB). (C and D) Rice protoplast cells expressing GFP only were untreated (C) or treated (D) with LMB (+LMB) for use as a negative control. Bars, 5 μm.

It has been reported that yeast NMD3 (ScNMD3) acts as a bridge between pre-60S subunits and the nuclear export factor CRM1/XPO1, and leptomycin B (LMB) could block this process (Ho *et al.*, 2000; Gadal *et al.*, 2001; Kressler *et al.*, 2010). To investigate whether OsNMD3 shares a similar export pathway with ScNMD3, the current work treated rice protoplasts transiently expressing OsNMD3GFP with LMB and examined the distribution of GFP signals. In contrast to the control cells expressing GFP only, which showed GFP signals in both the nucleus and cytoplasm (Fig. 2C, D), the OsNMD3GFP signals were retained in the nucleus after treatment (Fig. 2A, B). Taken together, these data indicate that OsNMD3 shuttles between the nucleus and cytoplasm via CRM1/XPO1 and functions as a nuclear export adaptor for 60S ribosomal subunits.

### Generation of rice plants expressing a dominant negative form of OsNMD3

**Fig. 3.**
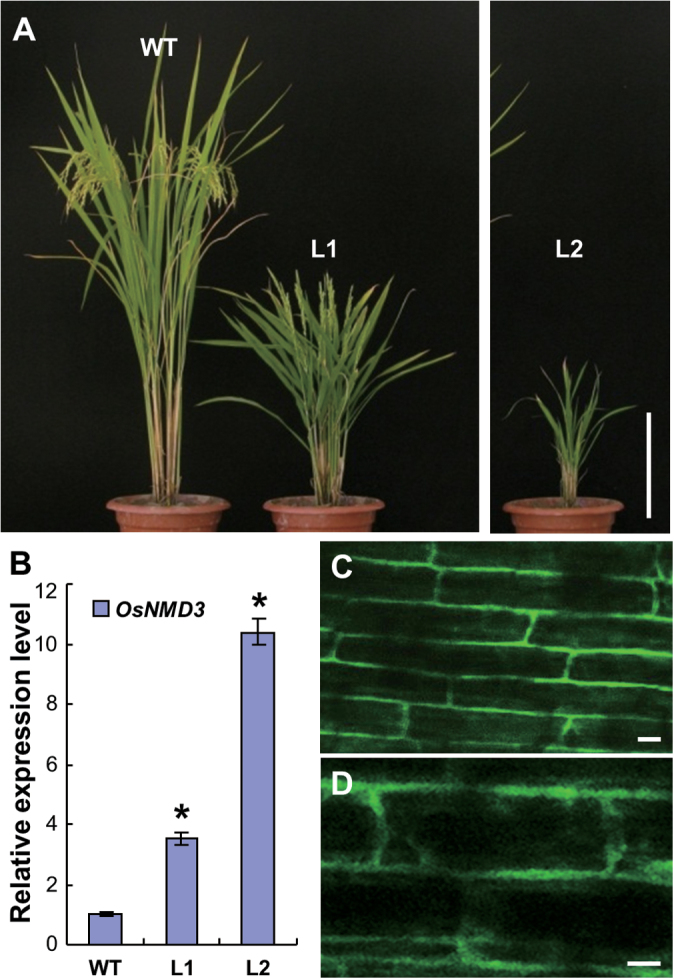
Expression of *OsNMD3* ^*ΔNLS*^ *GFP* affects plant growth. (A) Three-month-old wild-type and transgenic plants expressing *OsNMD3* ^*ΔNLS*^ *GFP*; L1 and L2 are two separate transgenic lines. (B) Relative expression of *OsNMD3* in wild-type and two transgenic lines; data are mean±SE (*n*=3, *P<0.01 by Student’s *t*-test). (C and D) OsNMD3^ΔNLS^GFP signals in the root cells (C); an enlarged image is also shown (D). Bars, 25cm (A) and 10 μm (C and D).

To genetically investigate the functions of OsNMD3 in ribosomal dynamics in rice plants, this work employed a dominant negative strategy by overexpression of the GFP-fused wild-type (*OsNMD3GFP*) and NLS (414–430 aa) truncated forms (*OsNMD3* ^*ΔNLS*^ *GFP*) in Nipponbare. The phenotypes of the transgenic plants were examined in the T2 generation. As shown in Fig. 3, the plants expressing *OsNMD3* ^*ΔNLS*^ *GFP* showed dwarfism. From two representative lines (L1 and L2), the plant size of L1 was obviously small compared with that of the wild type (Fig. 3A). The abnormality was more severe in L2, as the expression level of *NMD3* ^*ΔNLS*^ *GFP* was further increased in line 2 (L2, Fig. 3B). The L2 plants exhibited pleiotropic phenotypes, including dwarfism and sterility, and they rarely lived to maturity. An observation of the GFP signals in the root cells of these transgenic plants revealed that OsNMD3^ΔNLS^ was localized in the cytoplasm (Fig. 3C, D), consistent with the findings obtained with transient expression (Fig. 1C). However, the plants expressing *OsNMD3GFP* exhibited a wild-type appearance, although the expression level of *OsNMD3* in the transgenic plants was significantly upregulated. The GFP signals in these transgenic plants were found in the cytoplasm and nuclei (Supplementary Fig. S2).

### OsNMD3^ΔNLS^ alters the ribosome profile

**Fig. 4.**
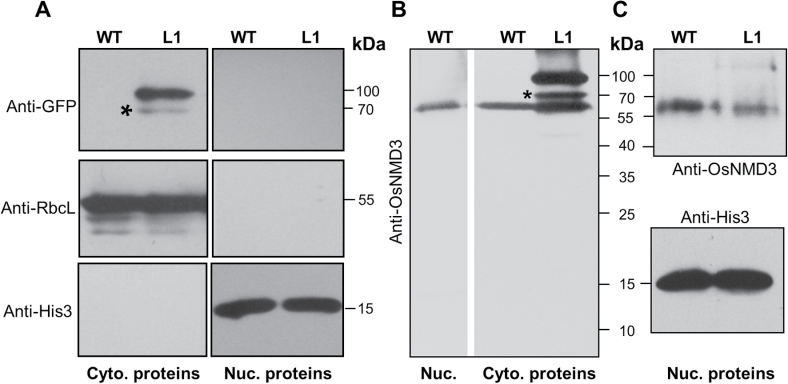
Immunodetecting OsNMD3 and OsNMD3^ΔNLS^ in wild-type and *OsNMD3* ^*ΔNLS*^ *GFP* transgenic plants. (A–C) Protein gel blotting of cytoplasmic and nuclear proteins extracted from wild-type (WT) and transgenic (L1) plants with the indicated antibodies. Cyto., cytoplasmic; nuc., nuclear. Asterisks indicate degraded OsNMD3^ΔNLS^GFP or nonspecific signals. Molecular weights are indicated at the right of each image.

To elucidate the molecular basis underlying the abnormalities observed in plants expressing *OsNMD3* ^*ΔNLS*^, several biochemical experiments were conducted. Because the L2 plants were sterile and it was difficult to collect enough material, the following biochemical analyses were performed in wild-type and L1 plants. First, the total proteins extracted from the wild-type and L1 plants were separated into cytoplasmic and nuclear fractions. The success of fractionation was demonstrated by probing both fractions with anti-RbcL and anti-histone3 antibodies, which are markers for the cytosol and nucleus, respectively (Fig. 4A). OsNMD3^ΔNLS^ proteins were detected in the cytosolic fraction with anti-GFP antibodies (Fig. 4A). To examine the endogenous level of OsNMD3, a specific antibody targeted against OsNMD3 was generated using a peptide containing the amino acid residues 415–518 as an antigen. This antibody produced a marked band with a molecular weight of 60kDa in the wild-type plants (Fig. 4B), which is consistent with the predicted molecular weight of OsNMD3, indicating the specificity of this antibody. This antibody labelled the endogenous OsNMD3 in the cytoplasmic and nuclear fractions of wild-type plants, and it labelled a majority of OsNMD3^ΔNLS^ in the cytoplasm of transgenic plants (Fig. 4B, C). In addition, overexpression of *OsNMD3* ^*ΔNLS*^ altered the distribution of endogenous OsNMD3 in the cytoplasm and nucleus (Fig. 4B, C). The band detected in the cytosolic fraction at a molecular weight of 70kDa might be a degraded OsNMD3^ΔNLS^GFP or nonspecific signal.

**Fig. 5.**
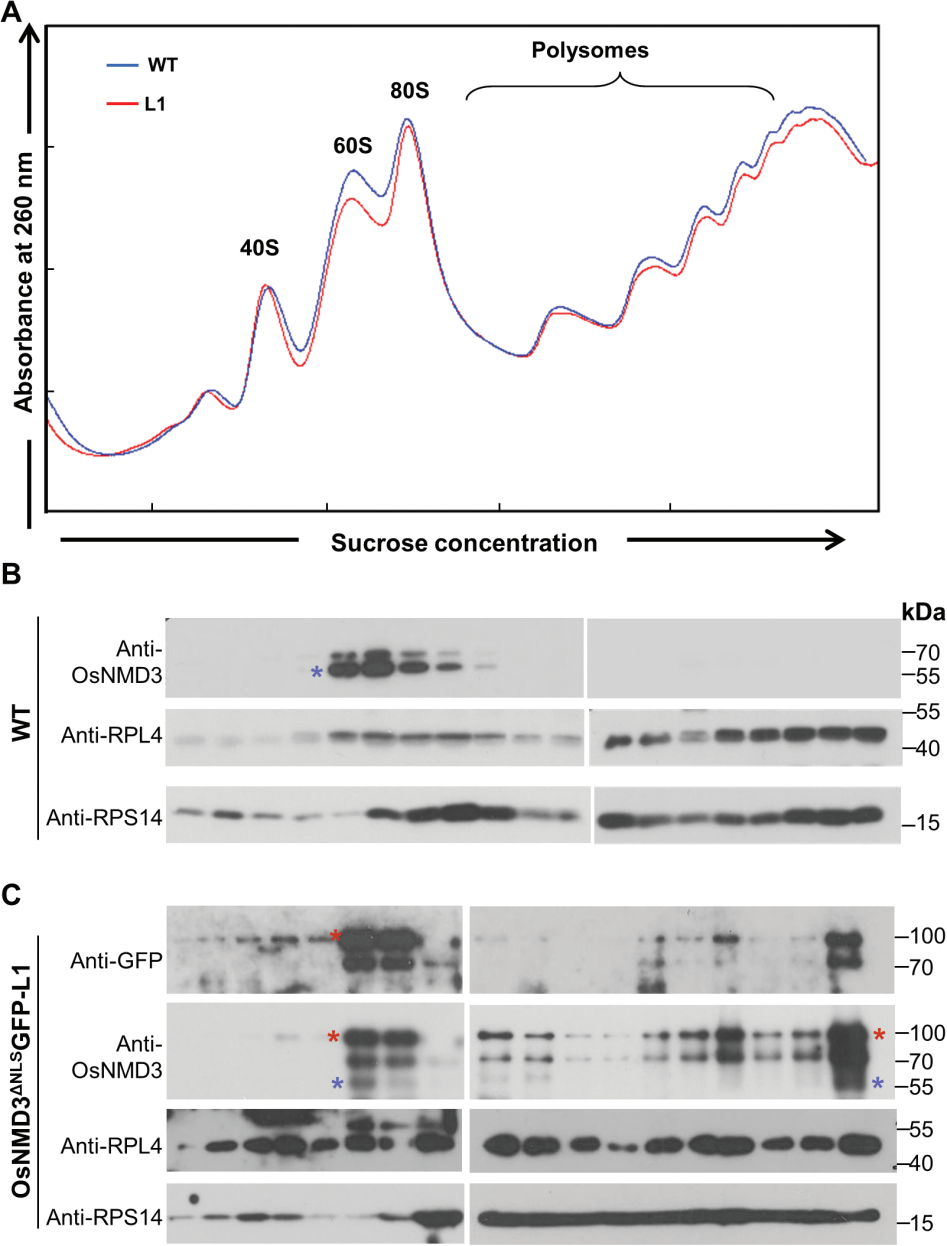
OsNMD3^ΔNLS^ interfered with the release of OsNMD3 from the 60S subunit. (A) Ribosomal extracts from wild-type and L1 plants (*OsNMD3* ^*ΔNLS*^ *GFP*) were fractionated on a 5–50% sucrose density gradient; *A*_260_ peaks representing free 40S and 60S subunits, 80S monosomes, and polysomes are indicated. (B and C) Fractions from the gradient shown in A were collected and subjected to immunoblotting with the indicated antibodies; RPL4 and RPS14 were used as the markers for the 60S and 40S subunits, respectively; red and blue asterisks indicate exogenous and endogenous OsNMD3, respectively (this figure is available in colour at *JXB* online).

This work further analysed the polysome profiles by ultracentrifugation of the ribosome particles from the wild-type and L1 plants on sucrose density gradients. As shown in Figure 5A, which illustrates one representative result of three independent replicates, there was a slight decrease in free 60S ribosomal subunits and polysomes in the L1 plants. The protein compositions of the gradient fractions were further determined by protein gel blotting. By probing the sucrose gradients of wild-type plants with anti-OsNMD3 antibodies, the majority of endogenous OsNMD3 was observed to cosediment with the free 60S subunits and was absent in the polysome fractions (Fig. 5B). The large ribosomal protein 4 (RPL4) and small ribosomal protein 14 (RPS14) were used as markers to monitor the sedimentation of 60S and 40S ribosomes, respectively (Fig. 5B). In the transgenic plants, the anti-GFP antibody recognized OsNMD3^ΔNLS^ in 60S subunits and polysomes, while anti-OsNMD3 antibodies labelled both the exogenous and endogenous OsNMD3 in the same fractions (Fig. 5C). The presence of OsNMD3 in polysomes suggested that overexpression of *OsNMD3* ^*ΔNLS*^ *GFP* interfered with the release of OsNMD3 from the 60S subunits. In addition, the distribution and abundance of RPL4 in the gradients was obviously altered (Fig. 5C). Therefore, OsNMD3^ΔNLS^ affected the release of OsNMD3 from 60S subunits and altered the ribosomal assembly.

### OsNMD3 and OsNMD3^ΔNLS^ interact with OsRPL10Ac1

**Fig. 6.**
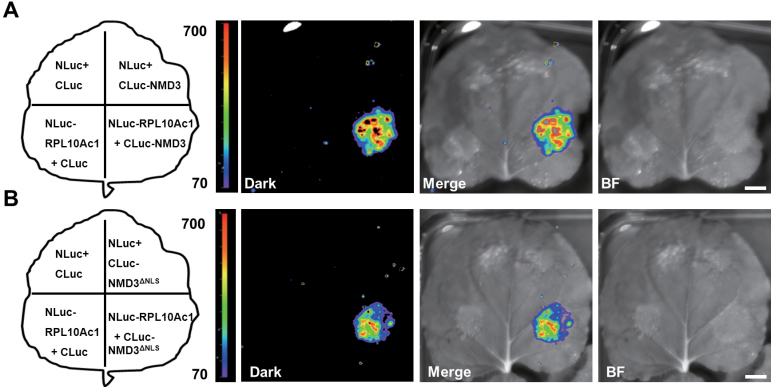
OsNMD3^ΔNLS^ interferes with the release of OsNMD3 from the 60S subunit. (A and B) Firefly luciferase complementation image showing the interaction of RPL10Ac1 with wild-type OsNMD3 (A) and OsNMD3^ΔNLS^ (B). BF, bright field. Bar, 1cm.

To obtain further evidence that OsNMD3 binds 60S subunits, this work determined which ribosomal proteins interact with this NMD3. RPL10 was reported to bind ScNMD3 and was thought to essential for its release from 60S subunits (Hedges *et al.*, 2005). Six RPL10 homologues are present in the rice genome. The interaction between OsNMD3 and these RPL10s was examined via a split-luciferase (split-LUC) complementation assay and yeast-two hybrid analysis. OsRPL10Ac1 (Os08g44450) was found to interact with OsNMD3 (Fig. 6A and Supplementary Fig. S3). Interestingly, deletion of the NLS did not affect the binding of OsNMD3 to OsRPL10Ac1 (Fig. 6B). This result led to the proposal that the abundant cytoplasmic OsNMD3^ΔNLS^ in L1 may interfere with the binding and release dynamics between OsNMD3 and 60S subunit.

### Protein synthesis efficiency is decreased in plants overexpressing OsNMD3^ΔNLS^

**Fig. 7.**
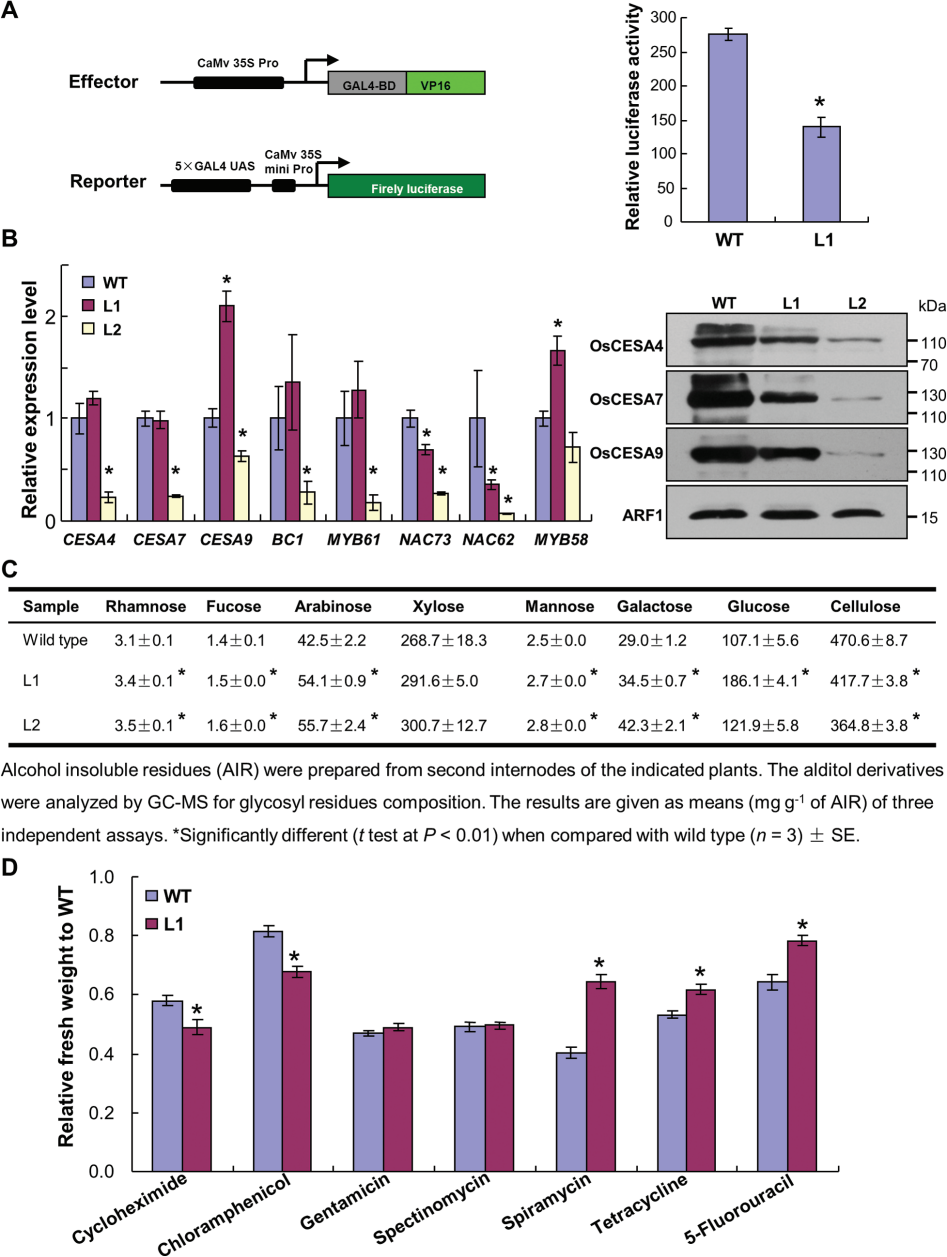
The translational efficiency of plants expressing *OsNMD3* ^*ΔNLS*^ *GFP* is reduced. (A) Transactivation assay of GAL4-VP16 in protoplast cells isolated from wild-type and L1 plants. (B) Relative expression levels of genes implicated in cell-wall biosynthesis in wild-type and two transgenic lines (left panel); data are mean±SE (*n*=3, *P<0.01 by Student’s *t*-test); protein blots were probed with rice CESA-specific antibodies; total membrane proteins were extracted from wild-type and two transgenic lines (right panel); molecular weights (kDa) are indicated. (C) Cell-wall composition of the internodes of the wild-type and two transgenic lines. (D) Pharmaceutical treatment of 4-d-old wild-type and L1 seedlings was performed in media containing different concentrations of antibiotics and chemicals for 6 d; relative freshweight was determined by considering the weight of untreated seedlings as the reference. Data are mean±SE (*n*=30, *P<0.01 by Student’s *t*-test) (this figure is available in colour at *JXB* online).

The inferior release of OsNMD3 may compromise the maturation of pre-60S particles, leading to aberrant ribosome assembly and low protein synthesis efficiency (Panse and Johnson, 2010; Karbstein, 2013). To evaluate this possibility in L1 plants, the current work expressed the GAL4-VP16 effector, a potent transcriptional activator (Sadowski *et al.*, 1988), and a reporter harbouring the firefly luciferase gene in protoplasts isolated from the wild-type and L1 plants and then examined the luciferase activity in these protoplasts. The significantly low level of luciferase activity in protoplasts derived from L1 plants suggested that protein synthesis in the L1 plants was disturbed (Fig. 7A). Cell-wall biosynthesis is a basic process involved in plant growth (Somerville *et al.*, 2004); thus, it was used as metric to determine whether protein synthesis efficiency was altered in the transgenic plants. At the RNA level, most of the examined cell-wall-related genes were downregulated in the L2 plants (Fig. 7B). Although the RNA levels of three *cellulose synthase* (*CESA*) genes were not decreased in the L1 plants, their protein levels were obviously decreased in L1 and dramatically decreased in L2 (Fig. 7B), which is consistent with their reduced cellulose content (Fig. 7C). The alterations in other noncellulosic sugars further suggested a broad change in cell-wall composition. Therefore, the reduced abundance of CESAs and cellulose content suggested that translation was compromised in the transgenic lines.

This work further employed pharmaceutical treatments to verify the above conclusion. Cycloheximide is an inhibitor of protein synthesis (Oleinick, 1977). The L1 plants showed moderate hypersensitivity to this chemical, indicating altered protein synthesis efficiency (Fig. 7D). To determine whether this deficiency was due to aberrant ribosomal structure, the wild-type and L1 seedlings were treated with a series of antibiotics and measured the relative freshweight after the treatments. The antibiotics applied for these pharmaceutical treatments are targeted to different ribosomal locations in prokaryotes (Rosado *et al.*, 2010). Compared with the wild-type, L1 seedlings were resistant or hypersensitive to specific antibiotics or remained unchanged (Fig. 7D and Supplementary Fig. S4). The distinct responses of L1 plants suggested that OsNMD3^ΔNLS^ caused a structural alteration in the ribosome. 5-Fluorouracil is an inhibitor of pre-60S subunit maturation (Fang *et al.*, 2004; Staley and Woolford, 2009). Incubation of the wild-type and L1 seedlings in media containing 5-fluorouracil inhibited plant growth. Measurement of the relative freshweight revealed that L1 plants were more resistant to 5-fluorouracil treatment compared with the wild-type (Fig. 7D and Supplementary Fig. S4).

Taken together, these data indicate that expression of *OsNMD3* ^*ΔNLS*^ interferes with ribosomal quality in terms of pre-60S subunit maturation, leading to a low translational efficiency.

### OsNMD3^ΔNLS^ affects the transcription of many ribosomal genes

**Fig. 8.**
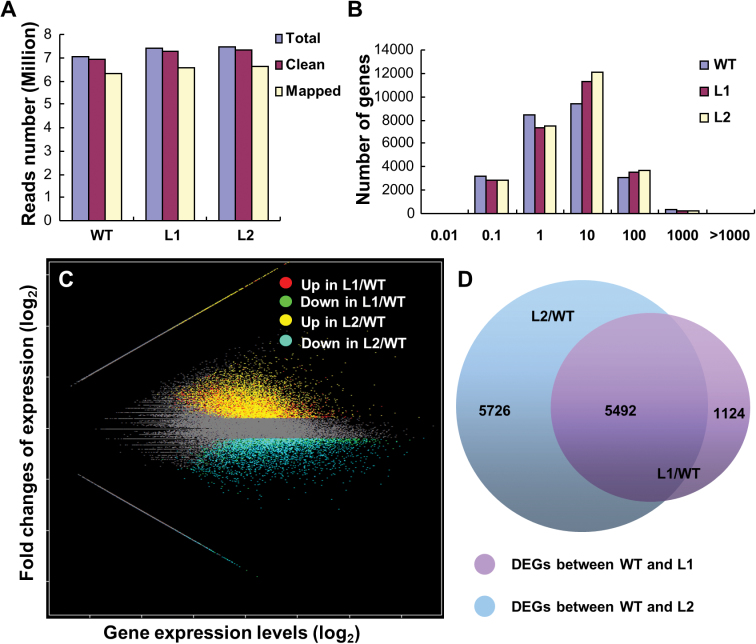
RNAseq of mRNA extracted from the internodes of wild-type plants and plants expressing the *OsNMD3* ^*ΔNLS*^ *GFP* transgene. (A) Reads from wild-type and two transgenic lines, indicating the quality of the RNAseq data. (B) Frequency of detected genes according to expression level. (C) Scatter plot showing the overall alterations in gene expression in the transgenic plants. (D) Numbers of genes with significantly altered expression levels between wild-type and transgenic plants (this figure is available in colour at *JXB* online).

Plants have mechanisms that coordinate protein synthesis and gene transcription, although they operate at different cellular sites (Chen *et al.*, 2012). The altered translational efficiency may have a feedback effect on gene expression. To determine the global changes in ribosome biogenesis, an Illumina RNA sequencing approach was used to investigate the global expression profiles of the wild-type, L1, and L2 plants. Because the major phenotype of the transgenic plants was dwarfism, internode mRNA was used for sequencing. Mapping the sequencing reads to rice reference cDNAs (MSU version 6.1) generated a total of 6.35, 6.56 and 6.60 million mapped reads from 6.94, 7.30, and 7.35 million clean reads of the wild-type, L1, and L2 plants, respectively (Fig. 8A). The sequencing quality was reliable because the number of detected genes was saturated and the reads were distributed uniformly at random (Supplementary Fig. S5). Of the 29 030 genes that were expressed in either wild-type or transgenic plants, 12 342 DEGs were detected, including 5351 (L1) and 7787 (L2) upregulated genes and 1265 (L1) and 3431 (L2) downregulated genes compared with the wild type (Fig. 8B, C and Supplementary Data S1 and S2). In addition, 5492 DEGs were consistently altered in both lines (Fig. 8D).

**Fig. 9.**
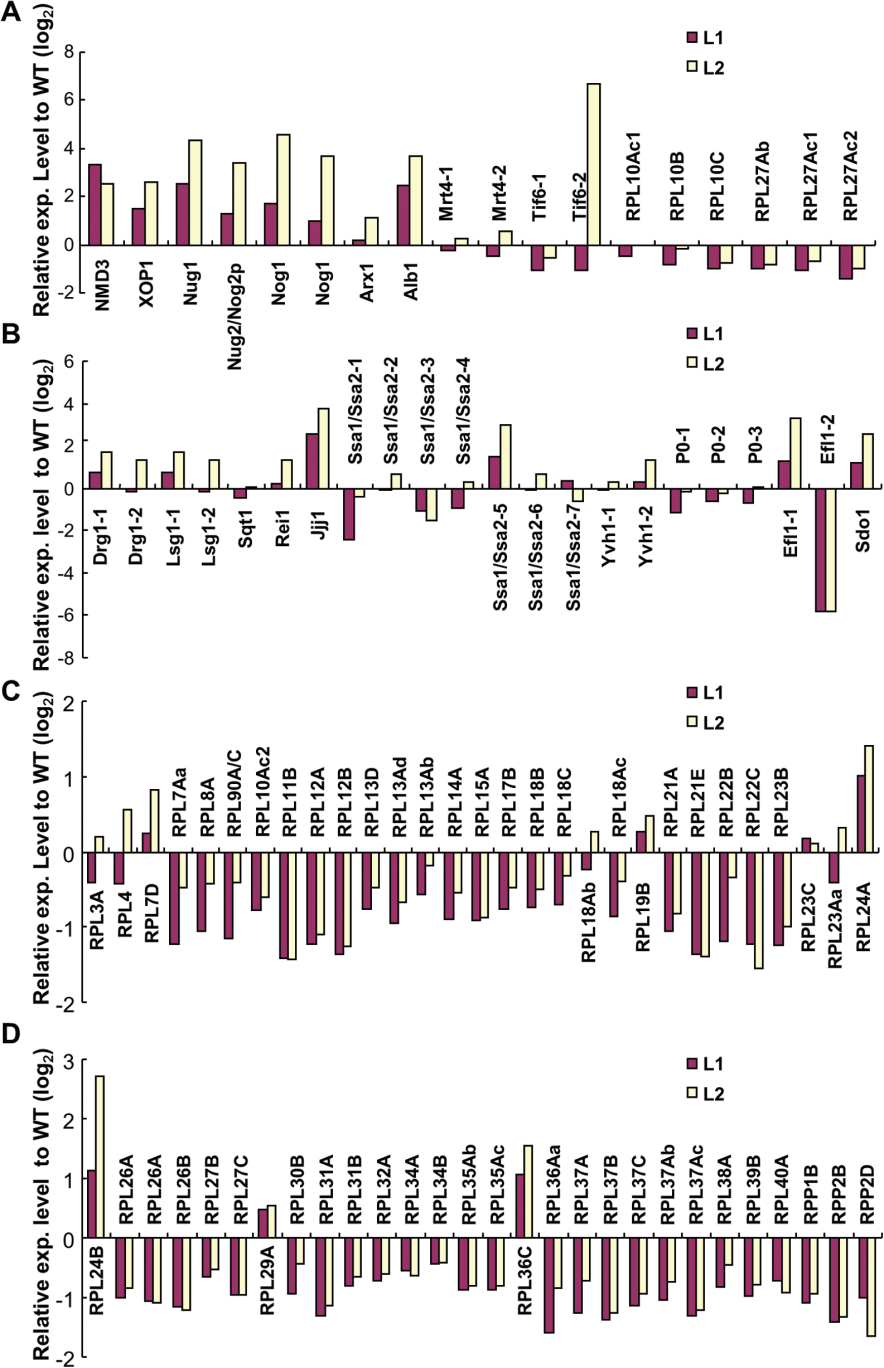
Expression levels of genes required for ribosome biogenesis in the plants expressing the *OsNMD3* ^*ΔNLS*^ *GFP* transgene. (A) Genes encoding *trans*-factors for 60S subunit export and biogenesis in nucleus. (B) Genes encoding *trans*-factors for 60S subunit cytoplasmic maturation. (C and D) Genes encoding ribosomal proteins of 60S subunits (this figure is available in colour at *JXB* online).

Based on the above data, this work found that the expression level of several genes required for 60S subunit biogenesis in nucleus/nucleolus was obviously altered (Fig. 9A). These factors included 60S subunit export adaptors, ATP/GTP-consuming enzymes, and RPLs. In addition, the expression level of genes required for cytoplasmic maturation was altered in the transgenic plants (Fig. 9B), indicating that the cytoplasmic maturation step was affected. During ribosomal biogenesis, loading RPs on the pre-ribosome subunits is essential for ensuring functionality (Karbstein, 2013). Therefore, the current work explored the expression level of many genes that encode RPs of the large subunit and found that most of them were downregulated (Fig. 9C, D and Supplementary Table S3). Therefore, expression of *OsNMD3* ^*ΔNLS*^ altered the expression profile of many genes implicated in ribosome biogenesis.

### Expression of OsNMD3^ΔNLS^ alters some agronomic traits

**Fig. 10.**
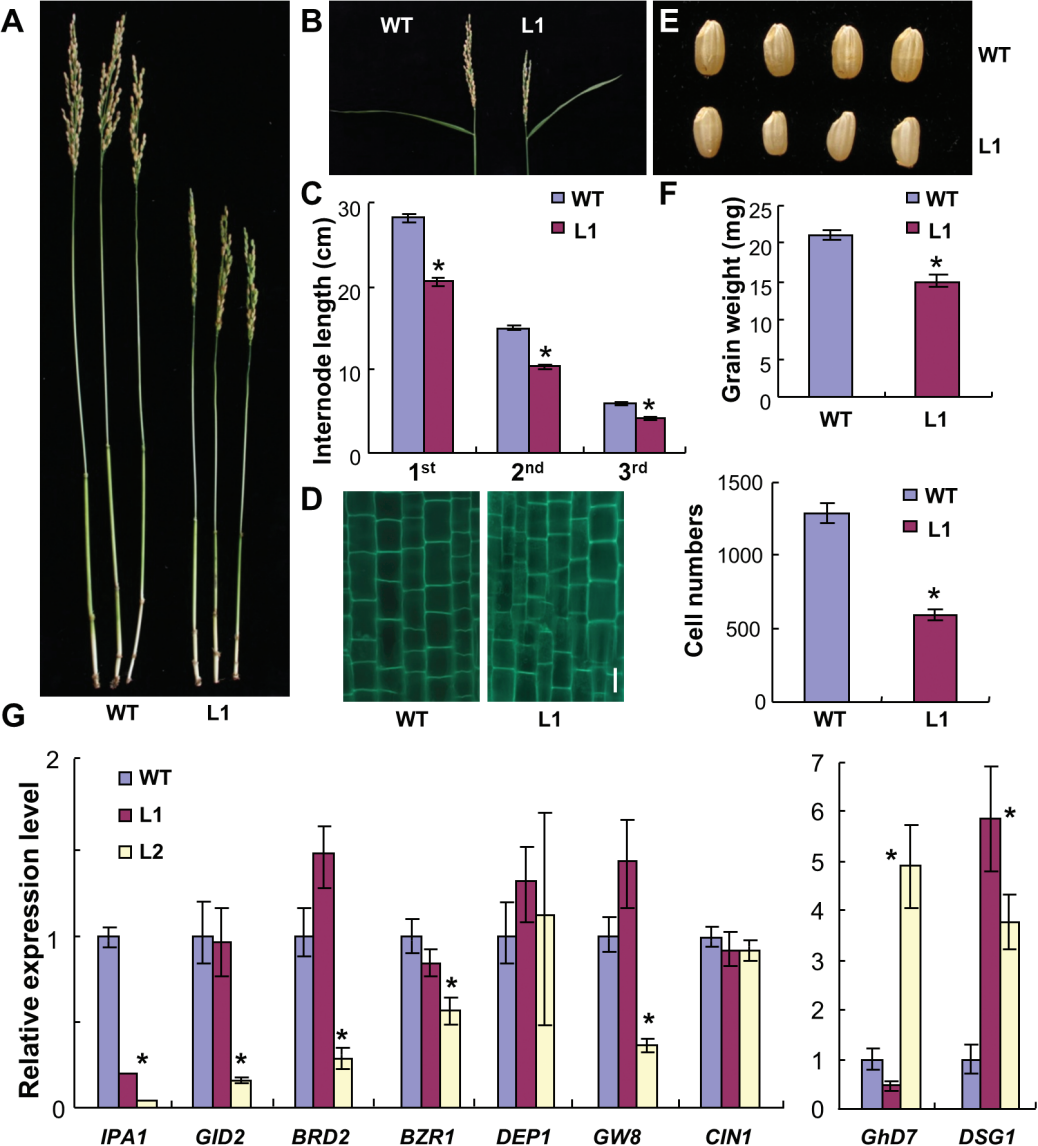
Examination of the phenotypes in plants expressing the *OsNMD3* ^*ΔNLS*^ *GFP* transgene. (A and B) Internodes and panicles of 5-month-old wild-type and L1 plants. (C) Comparison of internode length between 5-month-old wild-type and L1 plants; data are mean±SE (*n*=30, *P<0.01 by Student’s *t*-test). (D) Numbers of cells across the longitudinal axis of the third internode in wild-type and L1 plants; data are mean±SE (*n*=5, *P<0.01 by Student’s *t*-test). (E and F) Grain size and weight between the wild-type and L1 plants; data are mean±SE (*n*=3, *P<0.01 by Student’s *t*-test). (G) Relative expression of the indicated genes in wild-type and two transgenic lines; data are mean±SE (*n*=3, *P<0.01 by Student’s *t*-test) (this figure is available in colour at *JXB* online).

The aforementioned results prompted further examination of the effects of these aberrant 60S particles. Because the major phenotype of transgenic plants was dwarfism, the internode lengths of wild-type and L1 plants were compared, revealing a general length decrease in L1. The reduction mainly resulted from significantly decreased cell number based on analysis of longitudinal sections of internodes (Fig. 10A–D). Similar to the small panicle size (Fig. 10B), the grain size of L1 plants was also decreased, leading to reduced grain weight (Fig. 10E, F). Plant height, panicle and grain size, and grain weight are important agronomy traits that have been found to be controlled by specific quantitative trait loci (QTL) or genes. *IPA1* (*Ideal Plant Architecture1*), *GID2* (*Gibberellin-Insensitive Dwarf2*), *BRD2* (*Brassinosteroid-Deficient Dwarf2*), and *BZR1* (*Brassinazole-Resistant1*) are genes that modulate plant height (Ashikari *et al.*, 2005; Hong *et al.*, 2005; Bai *et al.*, 2007; Jiao *et al*.; 2010). qRT-PCR analysis showed that *IPA1* was significantly downregulated in both transgenic lines, whereas the expression of the other genes examined was repressed only in L2 (Fig. 10G). The reduced expression level of *IPA1* may be a primary effect of the aberrant ribosomal biogenesis in the transgenic plants. *DEP1* (*Dense and Erect Panicle1*), *GW8* (*Grain Width8*), *CIN1* (*Cell wall Invertase1*), and *GHD7* (*Grain number, plant height, and heading date7*), are the QTL or genes controlling panicle size, grain filling, grain size, and grain weight (Hirose *et al.*, 2002; Xue *et al.*, 2008; Huang *et al.*, 2009; Wang *et al.*, 2012). qRT-PCR revealed comparable as well as different expression levels (Fig. 10G), indicating that the phenotypes may not have resulted from changes in these particular genes. *DSG1* (*Delayed Seed Germination1*) is involved in stress responses (Park *et al.*, 2010). Upregulation of *DSG1* in both transgenic lines suggested that the transgenic plants were in a defensive state (Fig. 10G). The current work further employed KEGG enrichment analysis to reveal the affected pathways downstream of the above examined genes. Of tens of affected pathways, cell cycle, aminoacyl-tRNA biosynthesis, cytoskeleton, and stress responses were common between and ranked high in significance in both lines (Supplementary Tables S4 and S5). These data suggest that the compromised pre-60S particles may trigger translational incompetence of certain genes, which then alters the downstream gene expression.

Taken together, these data indicate that aberrant ribosomal subunits interfere with some basic pathways of plant development by controlling the translation efficiency of certain key regulators.

## Discussion

Ribosomes comprise the basic machinery that decodes genetic information into proteins. Although the understanding of the elaborate processes of eukaryotic ribosome modification, transport, and maturation has been greatly enhanced based on the findings achieved in yeasts and animals, relevant knowledge in plants is still very limited (Dresios *et al.*, 2006; Panse and Johnson, 2010; Karbstein, 2013). Recently, increasing studies on ribosome biogenesis have been performed in *Arabidopsis thaliana*, a dicotyledonous model plant. These studies were dedicated to elucidating the functions of RPs, which function in embryo biogenesis, leaf and flower development, vacuolar trafficking, and UV response (Tzafrir *et al.*, 2004; Pinon *et al.*, 2008; Yao *et al.*, 2008; Fujikura *et al.*, 2009; Falcone *et al*., 2010; Rosado *et al.*, 2010; Szakonyi and Byrne, 2011). In addition, several *trans*-factors involved in ribosome biogenesis have been characterized (Shi *et al.*, 2005; Horvath *et al.*, 2006; Griffith *et al.*, 2007; Byrne, 2009; Chen *et al.*, 2012). However, a complete understanding of the mechanism of ribosome biogenesis in higher plants is currently far from being achieved. Rice is a monocot model system and, compared with *Arabidopsis*, fewer components related to ribosome biogenesis have been investigated in rice (Im *et al.*, 2011). A comprehensive understanding of ribosome biogenesis in rice plants is valuable because it will provide a new platform for the improvement of agronomic traits in this important crop. The current work reports the functions of a highly conserved protein, OsNMD3, in nuclear export and cytoplasmic maturation of pre-60S subunits. Knowledge about this protein provides a pathway for further investigation of the mechanisms of ribosomal biogenesis in rice plants.

In plants, the process of ribosome biogenesis has remained largely unknown; thus, the exploration of a conserved protein that has been well studied in other organisms is an optimal way to gain insight into ribosomal dynamics. NMD3 is such a protein due to its importance and conservation throughout eukaryotic species (Johnson *et al.*, 2002; Zemp and Kutay, 2007; Chen *et al.*, 2012). Rice possesses one annotated NMD3 sequence with all representative motifs, including Cx_2_C repeats at the N-terminus as well as an NLS and NES at the C-terminus. Transient expression assays in rice protoplasts revealed that OsNMD3 shuttles between the nucleus and cytoplasm via CRM1/XPO1, demonstrating its conserved behaviour as a pre-60S nuclear export adaptor, as reported in other species. OsNMD3 was further found to interact with the 60S subunit via OsRPL10Ac1, and it co-sedimented with 60S and 80S ribosome components in ribosome profile analysis.

After being transported from the nucleus, NMD3 must be released from pre-60S particles. This process occurs in the cytoplasm and is an important step toward 60S ribosome maturation (Panse and Johnson, 2010; Karbstein, 2013). In eukaryotes, the conserved *trans*-factor NMD3 and eIF6/Tif6 bind to the joining interface with 40S subunit, which blocks premature translation. Release of NMD3 from pre-60S particles can trigger the following events: joining with 40S subunits, processing precursor rRNAs, and forming fully functional ribosomes. Therefore, this step is critical for controlling ribosomal quality for translation (Gartmann *et al.*, 2010; Sengupta *et al.*, 2010; Klinge *et al.*, 2011). RPL10 and the GTPase LSG1 have been implicated in the release of NMD3 (Hedges *et al.*, 2005). However, nothing thus far is known about how cytoplasmic maturation of 60S pre-particles occurs in plants. The current work found that OsNMD3^ΔNLS^ was trapped in the cytoplasm. Therefore, truncated OsNMD3 disturbs the cytoplasmic maturation process of 60S particles and affects translational efficiency, which could be further supported by the following findings: (1) both OsNMD3 and OsNMD3^ΔNLS^ bound to OsRPL10Ac1; surplus cytoplasmic OsNMD3^ΔNLS^ may therefore bind to OsRPL10Ac1 and disturb its normal interaction with endogenous OsNMD3; (2) overexpression of *OsNMD3* ^*ΔNLS*^ altered the ribosomal structure and interfered with the release of endogenous OsNMD3 from 60S subunits; the presence of OsNMD3^ΔNLS^ in polysomes indicated that cytoplasmic OsNMD3^ΔNLS^ might be loaded onto the ribosome by an unknown mechanism; and (3) transgenic plants showed a reduction in translational efficiency and altered ribosomal structure based on transactivation assays and pharmaceutical treatments with several antibiotics. The protein abundance of CESAs, which participate in cellulose biosynthesis, was significantly low in the OsNMD3^ΔNLS^ transgenic lines, providing solid evidence that OsNMD3^ΔNLS^ decreased the efficiency of protein synthesis. The observation of reduced cellulose content and altered sugar composition was similar to that reported in *Arabidopsis* plants overexpressing *AtNMD3* ^*ΔNES*^ (Chen *et al.*, 2012). However, the underlying mechanism differs because the latter was due to the obstruction of AtNMD3 nuclear export. Cell-wall biosynthesis is sensitive to the functionality of nascent ribosomes because these processes are likely to require rapid, abundant protein synthesis. Moreover, RNA sequencing was used to perform a genome-wide examination of the pathways affected by the aberrant 60S particles. The expression level of many factors involved in ribosomal biogenesis was significantly altered in the transgenic plants. This study thus pioneered the knowledge about ribosomal assembly in higher plants, although further studies are needed.

Ribosome-mediated phenotype control is an intriguing means of trait manipulation, especially in crops such as rice. Therefore, this work characterized the phenotypes that arose due to aberrant ribosome quality in the transgenic plants. The plants expressing *OsNMD3* ^*ΔNLS*^ exhibited pleiotropic phenotypes, such as dwarfism, reduced grain weight, and slow growth, which were attributed to alterations in cell morphogenesis and proliferation pathways, as revealed by RNA sequencing analysis. The expression levels of *IPA1*, *DSG1*, and other genes, which locate upstream of these pathways and control certain agronomic traits (Jiao *et al.*, 2010; Park *et al.*, 2010) were significantly altered, suggesting that translation of certain genes might be sensitive to ribosome quality. This study therefore may provide a new platform for improving agronomic traits by manipulating protein synthesis in rice plants.

## Supplementary material

Supplementary data are available at *JXB* online.

Supplementary Fig. S1. Bioinformatics analysis of NMD3 sequences from different species.

Supplementary Fig. S2. Expression of *OsNMD3GFP* in rice.

Supplementary Fig. S3. Interaction of OsNMD3 with the indicated RPLs.

Supplementary Fig. S4. Growth status of wild-type and *OsNMD3* ^*ΔNLS*^ *GFP* transgenic plants treated with antibiotics and chemicals.

Supplementary Fig. S5. Quality of RNAseq data of wild-type and two transgenic lines expressing *OsNMD3* ^*ΔNLS*^ *GFP*.

Supplementary Table S1. PCR primers.

Supplementary Table S2. qRT-PCR primers.

Supplementary Table S3. Alterations in expression of genes encoding ribosomal proteins according to RNAseq data for wild-type and transgenic plants.

Supplementary Table S4. Significant pathways in L1 plants detected by KEGG analysis.

Supplementary Table S5. Significant pathways in L2 plants detected by KEGG analysis.

Supplementary Data S1. Genes upregulated or downregulated 2-fold in RNAseq data of L1 compared with wild type.

Supplementary Data S2. Genes upregulated or downregulated 2-fold in RNAseq Data of L2 compared with wild type.

Supplementary Data
