# Supplementary material for: Retention of OsNMD3 in the cytoplasm disturbs protein synthesis efficiency and affects plant development in rice
Source: J Exp Bot. 2014 Apr 10;65(12):3055–69. doi: 10.1093/jxb/eru150 (PMC4071826; doi:10.1093/jxb/eru150)
Supplement: Supplementary Data [file supp_65_12_3055__index.html]

Retention of OsNMD3 in the cytoplasm disturbs protein synthesis efficiency and affects plant development in rice — Supplementary Data 

# Retention of OsNMD3 in the cytoplasm disturbs protein synthesis efficiency and affects plant development in rice

## Supplementary Data

Data files

**Files in this Data Supplement:**

- Supplementary Data - Supplementary Data
- Supplementary Data - Supplementary Data
- Supplementary Data - Supplementary Data
