# Supplementary material for: Retention of OsNMD3 in the cytoplasm disturbs protein synthesis efficiency and affects plant development in rice
Source: J Exp Bot. 2014 Apr 10;65(12):3055–69. doi: 10.1093/jxb/eru150 (PMC4071826; doi:10.1093/jxb/eru150)
Supplement: Supplementary Data [file supp_eru150_Supplementary_data.pdf]

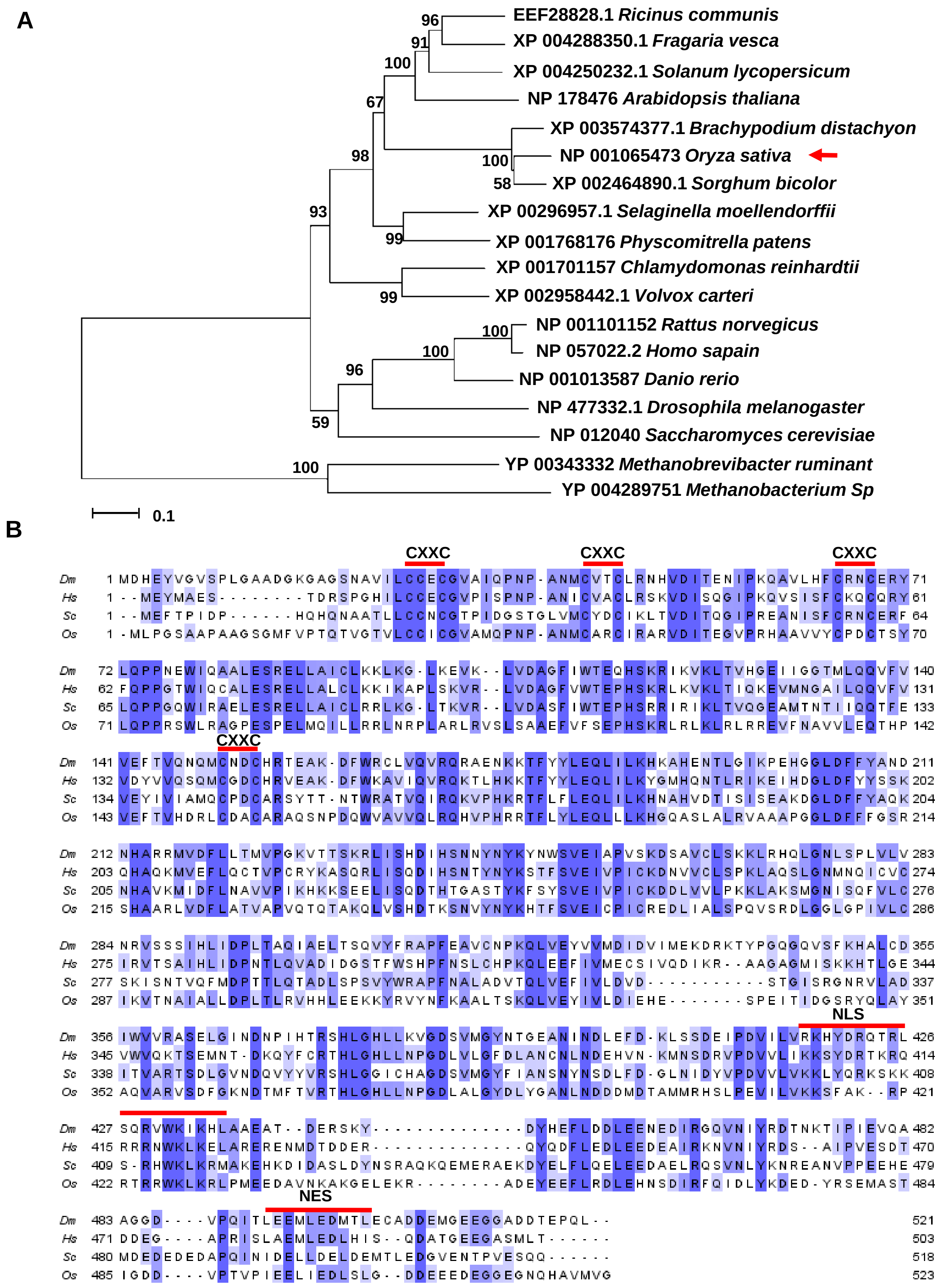
A
96
91
100
67
98
100
58
93
99
99
100
100
96
59
100
EEF28828.1 Ricinus communis
XP 004288350.1 Fragaria vesca
XP 004250232.1 Solanum lycopersicum
NP 178476 Arabidopsis thaliana
XP 003574377.1 Brachypodium distachyon
NP 001065473 Oryza sativa
XP 002464890.1 Sorghum bicolor
XP 00296957.1 Selaginella moellendorffii
XP 001768176 Physcomitrella patens
XP 001701157 Chlamydomonas reinhardtii
XP 002958442.1 Volvox carteri
NP 001101152 Rattus norvegicus
NP 057022.2 Homo sapain
NP 001013587 Danio rerio
NP 477332.1 Drosophila melanogaster
NP 012040 Saccharomyces cerevisiae
YP 00343332 Methanobrevibacter ruminant
YP 004289751 Methanobacterium Sp
0.1
B
CXXC
CXXC
CXXC
Dm 1 MDHEYVGVSPLGAADGKGAGSNAVILCCECGVAIQPNP-ANMCVTCLRNHVDITENIPKQAVLHFCRNCERY 71
Hs 1 --MEYMAES--------TDRSPGHILCCECGVPISPNP-ANICVACLRSKVDISQGIPKQVSISFCKQCQRY 61
Sc 1 --MEFTPIDP------HQHQNAATLLCCNCGTPIDGSTGLVMCYDCIKLTVDITQGIPREANISFCRNCERF 64
Os 1 -MLPGSAAPAAGSGMFVPTQTVGTVLCCICGVAMQPNP-ANMCARCIRARVDITEGVPRHAAVVYCPDCTSY 70
Dm 72 LQPPNEWIQAALESRELLAICLKKLKG-LKEVK--LVDAGFIWTEQHSKRIKVKLTVHGEIIGGTMLQQVFV 140
Hs 62 FQPPGTWIQCALESRELLALCLKKIKAPLSKVR--LVDAGFVWTEPHSKRLKVKLTIQKEVMNGAILQQVFV 131
Sc 65 LQPPGQWIRAELESRELLAICLRRLKG-LTKVR--LVDASFIWTEPHSRRIRIKLTVQGEAMTNTIIQQTFE 133
Os 71 LQPPRSWLRAGPESPELMQILLRRLNRPLARLRVSLSAAEFVFSEPHSKRLRLKLRLRREVFNAVVLEQTHP 142
CXXC
Dm 141 VEFTVQNQMCNDCHRTEAK-DFWRCLVQVRQRAENKKTFYYLEQLILKHKAHENTLGIKPEHGGLDFFYAND 211
Hs 132 VDYVVQSQMCGDCHRVEAK-DFWKAVIQVRQKTLHKKTFYYLEQLILKYGMHQNTLRIKEIHDGLDFYYSSK 202
Sc 134 VEYIVIAMQCPDCARSYTT-NTWRATVQIRQKVPHKRTFLFLEQLILKHNAHVDTISISEAKDGLDFFYAQK 204
Os 143 VEFTVHDRLCDACARAQSNPDQWVAVVQLRQHVPHRRTFLYLEQLLLKHGQASLALRVAAAPGGLDFFFGSR 214
Dm 212 NHARRMVDFLLTMVPGKVTTSKRLISHDIHSNNYNYKYNWSVEIAPVSKDSAVCLSKKLRHQLGNLSPLVLV 283
Hs 203 QHAQKMVEFLQCTVPCRYKASQRLISQDIHSNTYNYKSTFSVEIVPICKDNVVCLSPKLAQSLGNMNQICVC 274
Sc 205 NHAVKMIDFLNAVVPIKHKKSEELISQDTHTGASTYKFSYSVEIVPICKDDLVVLPKKLAKSMGNISQFVLC 276
Os 215 SHAARLVDFLATVAPVQTQTAKQLVSHDTKSNVYNYKHTFSVEICPICREDLIALSPQVSRDLGGLGPIVLC 286
Dm 284 NRVSSSIHLIDPLTAQIAELTSQVYFRAPFEAVCNPKQLVEYVVMDIDVIMEKDRKTYPGQGQVSFKHALCD 355
Hs 275 IRVTSAIHLIDPNTLQVADIDGSTFWSHPFNSLCHPKQLEEFIVMECSIVQDIKR--AAGAGMISKKHTLGE 344
Sc 277 SKISNTVQFMDPTTLQTADLSPSVYWRAPFNALADVTQLVEFIVLDVD-----------STGISRGNRVLAD 337
Os 287 IKVTNAIALLDPLTLRVHHLEEKKYRVYNFKAALTSKQLVEYIVLDIEHE-------SPEITIDGSRYQLAY 351
NLS
Dm 356 IWVVRASELGINDNPIHTRSHLGHLLKVGDSVMGYNTGEANINDLEFD-KLSSDEIPDVILVRKHYDRQTRL 426
Hs 345 VWVQKTSEMNT-DKQYFCRTHLGHLLNPGDLVLGFDLANCNLNDEHVN-KMNSDRVPDVVLIKKSYDRTKRQ 414
Sc 338 ITVARTSDLGVNDQVYYVRSHLGGICHAGDSVMGYFIANSNYNSDLFD-GLNIDYVPDVVLVKKLYQRKSKK 408
Os 352 AQVARVSDFGKNDTMFTVRTHLGHLLNPGDLALGYDLYGANLNDDDMDTAMMRHSLPEVILVKKSFAK--RP 421
Dm 427 SQRVWKIKHLAAEAT--DERSKY--------------DYHEFLDDLEENEDIRGQVNIYRDTNKTIPIEVQA 482
Hs 415 RRRNWKLKELARERENMDTDDER--------------QYQDFLEDLEEDEAIRKNVNIYRDS--AIPVESDT 470
Sc 409 S-RHWKLKRMAKEHKDIDASLDYNSRAQKQEMERAEKDYELFLQELEEDAELRQSVNLYKNREANVPPEEHE 479
Os 422 RTRRWKLKRLPMEEDAVNKAKGELEKR--------ADEYEEFLRDLEHNSDIRFQIDLYKDED-YRSEMAST 484
NES
Dm 483 AGGD----VPQITLEEMLEDMTLECADDEMGEEGGADDTEPQL-- 521
Hs 471 DDEG----APRISLAEMLEDLHIS--QDATGEEGASMLT------ 503
Sc 480 MDEDEDEDAPQINIDELLDELDEMTLEDGVENTPVESQQ------ 518
Os 485 IGDD----VPTVPIEELIEDLSLG--DDEEEDEGGEGNQHAVMVG 523

Supplementary Fig. S1. Bioinformatics analysis of NMD3 sequences from different species. (**A**) Neighbor-joining tree of rice NMD3 and its homologs from different species. The name of species is shown at the right. The numbers at each node represent the bootstrap support (percentage). The scale bar indicates genetic distance based on branch length. The red arrow indicats OsNMD3. (**B**) Alignment of OsNMD3 with its homologs. The conserved residues were shown in blue, and the conservation depends on darkness of the color. Red bars indicate the conserved Cx_2C repeats, a NLS, and a leucine-rich NES motifs. Numbers before the sequence indicate sites of the proteins. The accession numbers of proteins are shown as following: *Sc*, *S*. *cerevisiae* (GenBank 012040); *Dm*, *D*. *melanogaster* (GenBank 2661560); *Hs*, *H*. *sapain* (GenBank 057022); *Os*, *O*. *Sativa* (GenBank 001065473). Alignments were performed by using CLUSTALX.

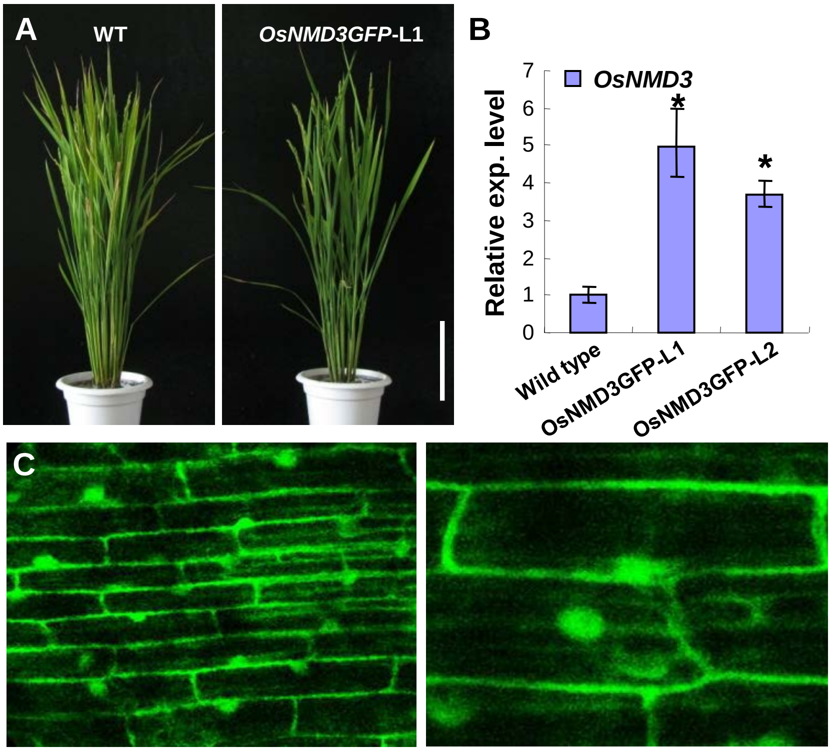

Supplementary Fig. S2. Expression of *OsNMD3GFP* in rice plants. (**A**) Two-month-old wild-type plants and plants expressing *OsNMD3GFP*. (**B**) Relative expression level of *OsNMD3* in wild-type and two transgenic lines; data are mean ± SE (n = 3, *P < 0.01 by Student's t-test). (**C**) *OsNMD3GFP* signals in the root cells of the relevant transgenic plants. Bars, 20 cm in (**A**) and 10 μm in (**C**).

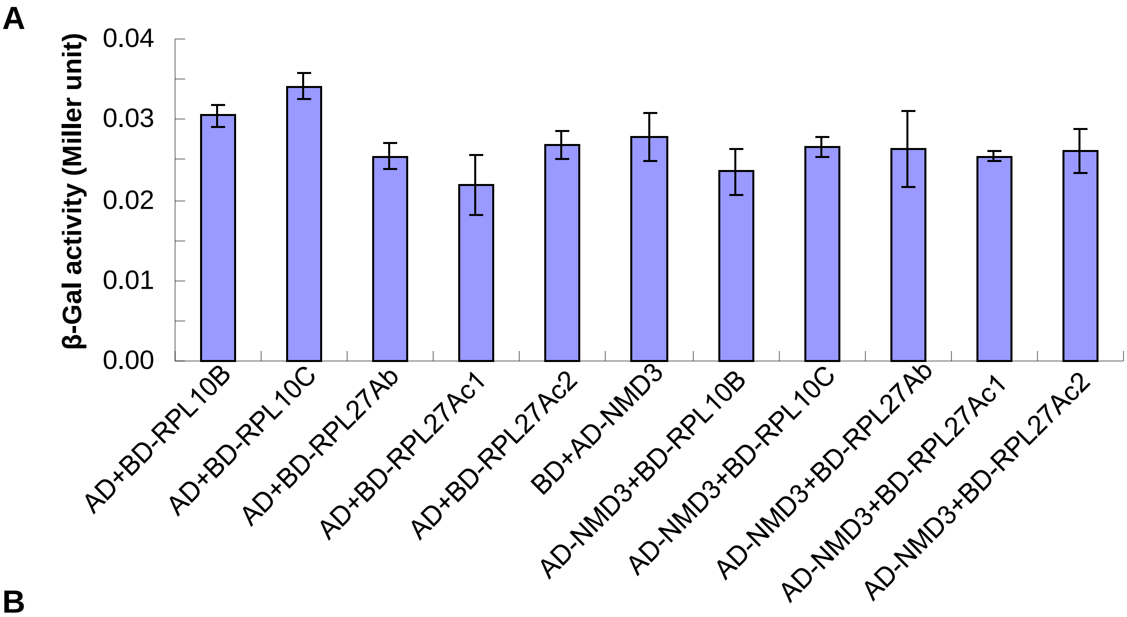

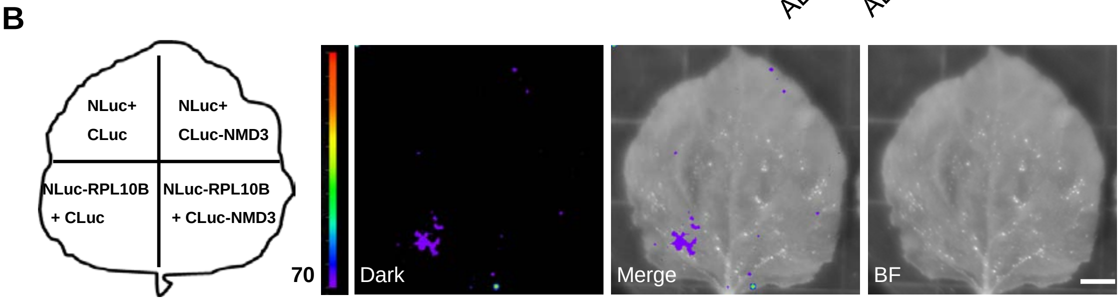

Supplementary Fig. S3. Interaction of OsNMD3 with the indicated RPLs. (**A**) *β*-galactosidase activity assay in yeast cells expressing *OsNMD3* and indicated *RPLs*. (**B**) Firefly luciferase complementation image showing the interaction of OsNMD3 with one representative RPL10 protein (RPL10B). Bars, 1 cm.

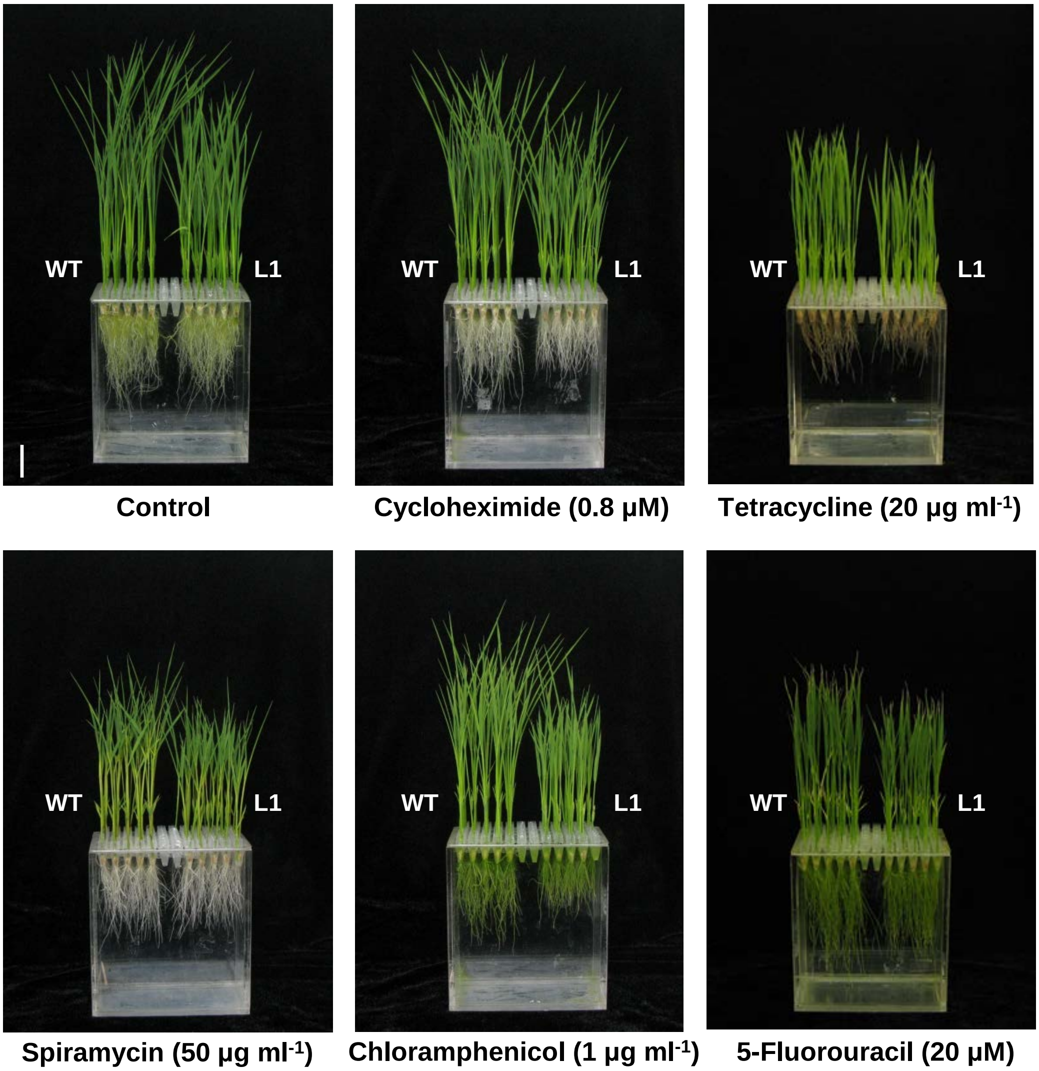

Supplementary Fig. S4. Growth status of wild-type and *OsNMD3*$^{\Delta NLS}$*GFP* transgenic plants treated with antibiotics and chemicals.

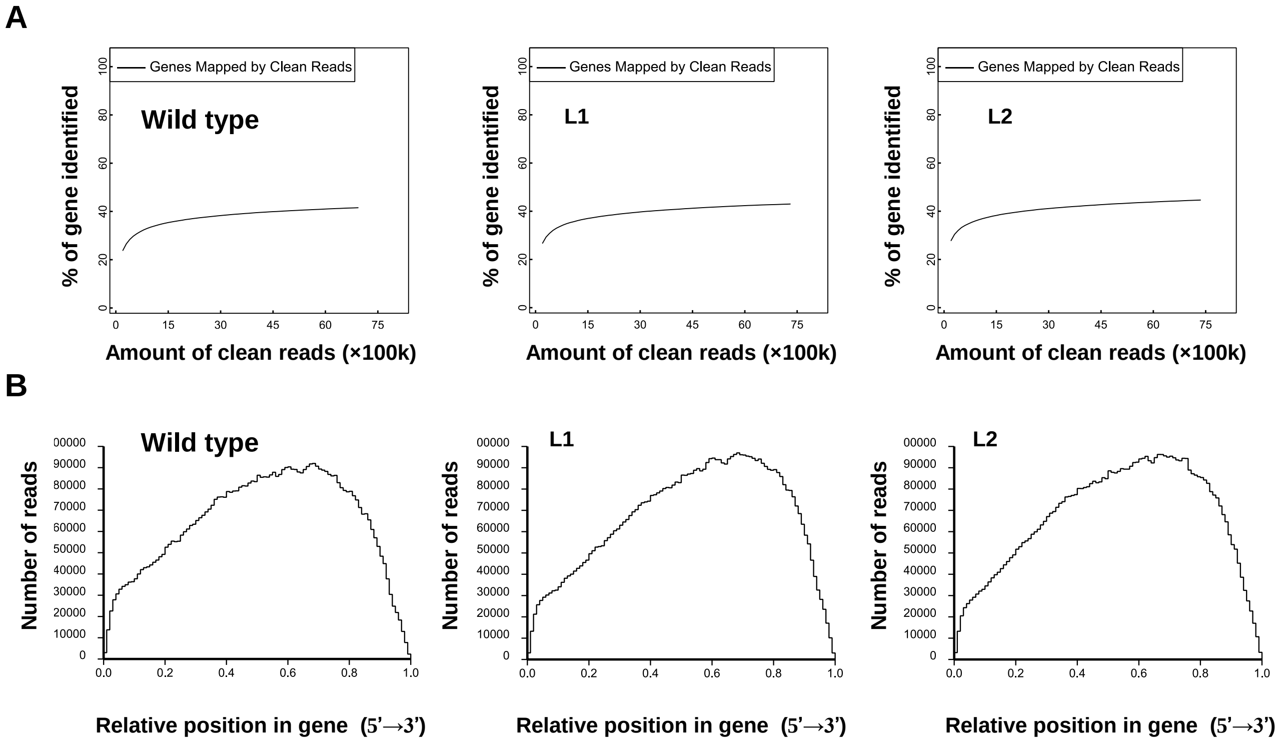

Supplementary Fig. S5. Quality of RNAseq data of wild-type and two transgenic lines expressing *OsNMD3*$^{\Delta NLS}$*GFP*. (**A**) Saturation analysis of the identified genes showed that the detected genes are saturated. (**B**) Random distribution of the reads in the reference genes.

Supplementary Tables

Supplementary Table S1. PCR primers.

Purpose	Orientation	Primer Sequence
Clone of *NMD3*	Forward	GGGGACAAGTTTGTACAAAAAAGCAGGCTCAATGCTGCCGGGGTCG
	Reverse 1	GGGGACCACTTTGTACAAGAAAGCTGGGTATCCCACCATAACAGCATGTTG
	Reverse 2	GGGGACCACTTTGTACAAGAAAGCTGGGTATCATCCCACCATAACAGCATG
Clone of *NMD3*$^{\Delta NES}$	Forward	CTCCTCATCATCACCCGGCACAGTGGGAACATCATCACC
	Reverse	GTTCCCACTGTGCCGGGTGATGATGAGGAGGAGGATGAAG
Clone of *NMD3*$^{\Delta NLS}$	Forward	GAAGTCATTCTTGTTCTACCAATGGAGGAAGATGCTGTAAAT
	Reverse	TTCCTCCATTGGAAGAACAAGAATGACTTCAGGCAGACTG
Antibody of NMD3	Forward	TAGAATTCAAGAAGAGCTTCGCCAAGAG
	Reverse	TCTCGAGATGTTGGTTGCCTTCCCCAC
Clone of *RPL10Ac1*	Forward	GGGGACAAGTTTGTACAAAAAAGCAGGCTCAATGAGTAAGTTGCAGAGCGA
	Reverse 1	GGGGACCACTTTGTACAAGAAAGCTGGGTATTAGAACACCCTGATTGGTT
	Reverse 2	GGGGACCACTTTGTACAAGAAAGCTGGGTAGAACACCCTGATTGGTTTCC
Clone of *RPL10B*	Forward	GGGGACAAGTTTGTACAAAAAAGCAGGCTCAATGGGGAGGAGACCTGCAAG
	Reverse 1	GGGGACCACTTTGTACAAGAAAGCTGGGTACTAAGCGGCTGAGAGGAAGG
	Reverse 2	GGGGACCACTTTGTACAAGAAAGCTGGGTAAGCGGCTGAGAGGAAGG
Clone of *RPL10C*	Forward	GGGGACAAGTTTGTACAAAAAAGCAGGCTCAATGGGGAGAAGGCCTGCTAG
	Reverse 1	GGGGACCACTTTGTACAAGAAAGCTGGGTACTAGGCGGAAGCTTGAATGG
	Reverse 2	GGGGACCACTTTGTACAAGAAAGCTGGGTAGGCGGAAGCTTGAATGG
Clone of *RPL27Ab*	Forward	GGGGACAAGTTTGTACAAAAAAGCAGGCTCAATGACGACGAGCCTGAGGAA
	Reverse 1	GGGGACCACTTTGTACAAGAAAGCTGGGTaCTAGGCGGTGAGCACGACGG
	Reverse 2	GGGGACCACTTTGTACAAGAAAGCTGGGTAGGCGGTGAGCACGACGG
Clone of *RPL27Ac1*	Forward	GGGGACAAGTTTGTACAAAAAAGCAGGCTCAATGACAACGCGCTTCAAGAAG A
	Reverse 1	GGGGACCACTTTGTACAAGAAAGCTGGGTACTAGGCGGTGAGGAGGACGG

	Reverse 2	GGGGACCACTTTGTACAAGAAAGCTGGGTAGGCGGTGAGGAGGACGG
Clone of *RPL27Ac2*	Forward	GGGGACAAGTTTGTACAAAAAAGCAGGCTcaATGACGACGAGGTTCAAGAAGAAC

Supplementary Table S2. qRT-PCR primers.

Genes	Forward primer sequence	Reverse primer sequence
UBQ5	ACCACTTCGACCGCCACTACT	ACGCCTAAGCCTGCTGGTT
NMD3	AGCACAACTCTGATATTAG	GGAACATCATCACCAATA
IPA1	TAAGGTGACTGGCGATTA	ACAGCATTAACACTGATACTT
DEP1	GTAACCCATGCTGTCTCA	TTCAACCTCGTCTCATAGC
CIN1	TTGCTGAGGATGATTGGA	AACAACTCGTGATACAACAA
GID2	CAGTTGTGAGCCTTATCT	TGAATCAAGAACCATCGT
BRD2	TTGTAATCCTGTCATCGT	AAGTTCACCTCATCTGAT
BZR1	TAGCCTTAGGTTAATCTTGTTG	ACAGTGAAGCCGTGAATA
DSG1	TGTCTGAACATCTGAATT	GCTGTATAGTGGTAGTATC
GW8	AGGAGTTTGATGAGGCCAAG	GCGTGTAGTATGGGCTCTCC
Ghd7	AGGTGCTACGAGAAGCAAATCC	GGGCCTCATCTCGGCATAG
CESA4	CTCCGAGACCACCACCACCAAC	ACCCATCGTCTTCGTCGCATTAG
CESA7	AAGCCATGCGGGGTCTCGTG	CATCCATCCGGTCATCCCTCTTG
CESA9	ATCGCGCTCTTCATCTCCATCTTC	ACTGCTCGTTCCTCCACCACTCC
BC1	CTTTGAAATTGCCTGATAGA	AAAGTTTGTGGTGTGATTT
MYB61	ATATCTTGAGCCACTTGT	ATCCTCTTGGTGATTCAG
NAC73	CAGGGCAGGTATATGATG	CACTCCTTATTGTCCAGTAA
NAC62	TCTTAACCTTACATTCATCCATCA	GCATCATCAGGACCTCTTC
MYB58	GTAGTAGAGACACAACAC	ATATAGCACTTCTCATTGG

Supplementary Table S3. Alterations in expression of genes encoding the ribosomal proteins according to RNAseq data for wild-type and transgenic plants.

Name	Gene ID	Gene length	Wild Type	L1	L2	log2 Ratio (L1/WT)	FDR	log2 Ratio (L2/WT)	FDR
RPL3A	LOC_Os11g06750	1607	382.19	287.04	438.28	-0.41	6.85E-30	0.20	2.82E-09
RPL3B	LOC_Os12g07010	1489	746.16	315.46	454.42	-1.24	0.00E+00	-0.72	2.21E-143
RPL4	LOC_Os07g08330	1723	195.23	145.93	288.79	-0.42	3.71E-17	0.56	0.00E+00
RPL6C	LOC_Os04g39700	974	185.03	118.44	188.25	-0.64	6.94E-20	0.02	7.27E-01
RPL7B	LOC_Os08g13690	1269	1112.48	402.55	604.43	-1.47	0.00E+00	-0.88	1.09E-259
RPL7D	LOC_Os04g51630	1674	103.20	123.36	182.08	0.26	5.67E-05	0.82	1.49E-11
RPL7Aa	LOC_Os08g23710	1205	603.89	259.43	432.99	-1.22	5.05E-229	-0.48	1.84E-46
RPL8A	LOC_Os12g38000	1130	624.15	300.91	462.72	-1.05	1.90E-175	-0.43	3.12E-37
RPL90A/C	LOC_Os09g31180	1490	384.36	172.28	290.04	-1.16	2.83E-166	-0.41	3.06E-27
RPL10B	LOC_Os05g07700	1669	622.74	353.14	555.25	-0.82	1.94E-169	-0.17	1.14E-09
RPL10C	LOC_Os11g11390	1059	340.52	170.02	196.41	-1.00	4.07E-83	-0.79	2.48E-56
RPL10Ac	LOC_Os08g44380	1910	93.84	139.40	238.32	0.57	3.64E-11	1.34	2.52E-11
RPL10Ac	LOC_Os08g44450	1276	201.98	143.85	194.73	-0.49	1.97E-17	-0.05	3.95E-01
RPL10Ac	LOC_Os02g21660	1478	335.49	197.50	221.59	-0.76	2.80E-72	-0.60	3.20E-47
RPL11B	LOC_Os05g11710	1029	320.45	186.90	199.65	-0.78	9.56E-50	-0.68	7.68E-40
RPL11B	LOC_Os01g10820	549	462.79	174.43	171.07	-1.41	2.21E-100	-1.44	2.57E-103
RPL12A	LOC_Os02g47140	984	375.24	161.12	174.96	-1.22	1.04E-116	-1.10	4.45E-99
RPL12B	LOC_Os04g50990	898	459.60	180.10	192.60	-1.35	7.30E-153	-1.25	4.14E-136
RPL13D	LOC_Os06g02510	1284	274.00	162.44	197.88	-0.75	2.62E-50	-0.47	4.82E-22
11669.m03747	LOC_Os03g37970	1150	738.98	303.02	501.70	-1.29	9.26E-291	-0.56	4.53E-71
RPL13Ad	LOC_Os03g54890	1164	414.59	214.78	262.13	-0.95	2.03E-101	-0.66	8.43E-55
RPL13Ab	LOC_Os07g01870	1298	137.06	92.80	120.92	-0.56	1.47E-15	-0.18	8.24E-03
RPL14A	LOC_Os02g40880	926	541.40	291.83	372.69	-0.89	6.36E-95	-0.54	1.20E-39
RPL15A	LOC_Os03g40180	1145	366.83	196.21	222.32	-0.90	2.59E-81	-0.72	1.05E-55
RPL15A	LOC_Os05g19370	1156	153.89	81.87	83.74	-0.91	2.58E-35	-0.88	2.20E-33
RPL17A	LOC_Os09g08430	950	399.85	409.91	786.85	0.04	5.31E-01	0.98	0.00E+00
RPL17B	LOC_Os08g41810	552	447.04	265.85	321.73	-0.75	4.01E-35	-0.47	5.28E-16
RPL18C	LOC_Os07g47780	901	58.75	43.50	61.80	-0.43	1.02E-03	0.07	6.02E-01
RPL18A	LOC_Os07g47780	901	58.75	43.50	61.80	-0.43	1.02E-03	0.07	6.02E-01
RPL18B	LOC_Os05g06310	1008	453.40	272.36	322.38	-0.74	2.38E-62	-0.49	7.66E-31
RPL18C	LOC_Os03g22180	892	271.44	167.37	218.47	-0.70	1.88E-30	-0.31	6.71E-08
RPL18Ab	LOC_Os01g54870	933	291.88	248.65	352.58	-0.23	2.84E-05	0.27	3.47E-08
RPL18Ac	LOC_Os01g47660	897	390.73	215.47	298.23	-0.86	1.69E-62	-0.39	8.45E-16
RPL19A	LOC_Os03g21940	1123	333.23	304.20	418.44	-0.13	6.02E-03	0.33	0.00E+00
RPL19B	LOC_Os03g38260	939	145.63	174.78	203.28	0.26	2.70E-04	0.48	3.19E-12
RPL21A	LOC_Os10g32820	495	790.53	380.80	449.61	-1.05	7.32E-98	-0.81	1.01E-63
RPL21E	LOC_Os03g04750	944	598.05	232.59	227.62	-1.36	2.17E-211	-1.39	2.05E-218
RPL22B	LOC_Os07g47710	950	310.06	136.19	244.21	-1.19	2.45E-89	-0.34	5.46E-11
RPL22C	LOC_Os03g22340	844	437.29	188.22	149.57	-1.22	4.01E-116	-1.55	2.82E-167
RPL23A	LOC_Os03g04590	1093	151.53	107.44	144.19	-0.50	7.37E-12	-0.07	3.47E-01

RPL23B	LOC_Os10g32920	650	699.80	298.33	351.99	-1.23	1.62E-145	-0.99	9.25E-103
RPL23C	LOC_Os02g56960	782	409.36	467.01	442.35	0.19	6.36E-05	0.11	2.26E-02
RPL23Aa	LOC_Os04g42270	996	691.29	518.64	859.10	-0.41	1.17E-33	0.31	8.80E-11
RPL23Ab	LOC_Os01g24690	923	392.31	344.05	713.31	-0.19	7.24E-05	0.86	0.00E+00
RPL24A	LOC_Os01g59990	1018	507.14	343.87	425.93	-0.56	1.13E-43	-0.25	1.16E-10
RPL24A	LOC_Os05g40820	990	162.26	255.26	346.66	0.65	0.00E+00	1.10	0.00E+00
RPL24A	LOC_Os07g12250	815	73.30	147.41	194.16	1.01	2.09E-12	1.41	1.14E-12
RPL24B	LOC_Os01g33050	1023	30.66	67.13	202.07	1.13	1.41E-12	2.72	1.27E-12
RPL24B	LOC_Os07g19190	1193	13.63	28.18	79.13	1.05	4.40E-09	2.54	0.00E+00
RPL26A	LOC_Os12g05430	823	239.95	120.23	133.56	-1.00	1.94E-45	-0.85	1.06E-34
RPL26A	LOC_Os11g05370	998	100.10	47.90	47.29	-1.06	7.76E-26	-1.08	1.07E-26
RPL26B	LOC_Os01g04730	705	332.61	149.62	142.30	-1.15	5.15E-68	-1.22	7.17E-75
RPL27B	LOC_Os02g18380	767	399.41	253.24	276.40	-0.66	1.30E-34	-0.53	5.80E-24
RPL27C	LOC_Os10g41470	974	226.78	117.29	116.63	-0.95	5.70E-47	-0.96	1.00E-47
RPL27Ab	LOC_Os07g42170	802	208.99	104.50	116.91	-1.00	7.59E-39	-0.84	3.63E-29
RPL27Ac	LOC_Os03g29460	791	479.60	233.26	304.22	-1.04	7.28E-93	-0.66	9.80E-43
RPL27Ac	LOC_Os02g07890	770	371.99	139.06	186.59	-1.42	1.63E-114	-1.00	1.81E-65
RPL29A	LOC_Os05g28750	183	283.84	394.42	413.54	0.47	2.27E-05	0.54	5.57E-07
RPL29B	LOC_Os01g19840	551	199.38	151.24	178.58	-0.40	7.85E-06	-0.16	7.66E-02
RPL30A	LOC_Os01g16890	1193	607.74	194.46	219.54	-1.64	0.00E+00	-1.47	2.35E-303
RPL30B	LOC_Os05g41110	822	469.18	244.43	344.52	-0.94	4.83E-80	-0.45	6.07E-22
RPL31A	LOC_Os02g48660	734	1074.83	435.90	492.63	-1.30	3.50E-275	-1.13	2.79E-218
RPL31B	LOC_Os06g21480	816	90.30	51.75	57.45	-0.80	2.29E-12	-0.65	4.46E-09
RPL31C	LOC_Os08g39500	722	44.82	38.40	76.90	-0.22	2.24E-01	0.78	2.92E-09
RPL32A	LOC_Os08g41300	723	881.85	538.84	576.92	-0.71	9.73E-82	-0.61	6.02E-63
RPL32B	LOC_Os09g32520	752	78.34	66.53	55.96	-0.24	6.72E-02	-0.49	7.53E-05
RPL34A	LOC_Os09g24690	706	341.54	235.85	220.73	-0.53	4.62E-19	-0.63	1.43E-25
RPL34B	LOC_Os08g33920	757	293.76	216.59	219.18	-0.44	1.24E-12	-0.42	5.07E-12
RPL34C	LOC_Os08g06040	693	210.25	229.23	284.21	0.12	1.12E-01	0.43	3.07E-11
RPL35B	LOC_Os02g30050	660	696.24	341.61	670.05	-1.03	4.14E-110	-0.06	2.16E-01
RPL35Ab	LOC_Os05g48220	824	122.25	66.91	69.71	-0.87	8.51E-19	-0.81	6.56E-17
RPL35Ac	LOC_Os05g48220	824	122.25	66.91	69.71	-0.87	8.51E-19	-0.81	6.56E-17
RPL36A	LOC_Os05g38520	784	1575.04	647.31	792.44	-1.28	0.00E+00	-0.99	3.81E-277
RPL36C	LOC_Os01g62350	620	46.04	96.63	134.97	1.07	2.48E-13	1.55	3.78E-13
RPL36Aa	LOC_Os07g26740	318	300.07	99.21	166.54	-1.60	8.34E-46	-0.85	2.44E-17
RPL37A	LOC_Os08g03450	534	423.27	177.54	258.87	-1.25	7.88E-75	-0.71	1.90E-29
RPL37B	LOC_Os02g56990	822	288.09	111.26	119.51	-1.37	4.25E-90	-1.27	6.58E-80
RPL37C	LOC_Os02g02130	285	241.65	110.70	125.75	-1.13	1.08E-19	-0.94	5.00E-15
RPL37Ab	LOC_Os01g48770	853	522.95	254.98	314.37	-1.04	1.86E-108	-0.73	9.01E-61
RPL37Ac	LOC_Os05g48320	682	442.86	180.36	190.03	-1.30	4.68E-105	-1.22	1.18E-95
RPL38A	LOC_Os07g36250	1790	5.38	6.41	7.69	0.25	4.48E-01	0.52	5.56E-02
RPL38A	LOC_Os11g24610	1058	410.01	231.47	299.88	-0.82	4.22E-72	-0.45	3.85E-25
RPL39A	LOC_Os06g08320	154	542.03	488.36	576.61	-0.15	1.76E-01	0.09	4.01E-01
RPL39B	LOC_Os02g55370	433	273.26	137.99	160.00	-0.99	5.64E-27	-0.77	3.29E-18

RPL40A	LOC_Os09g39500	780	401.90	244.93	213.74	-0.71	6.70E-41	-0.91	7.67E-62
RPL40A	LOC_Os03g15370	1113	0.60	0.14	91.57	-2.06	3.11E-01	7.26	9.91E-05
RPP0A	LOC_Os12g03880	1272	74.23	49.23	63.65	-0.59	1.47E-09	-0.22	2.00E-02
RPP0B	LOC_Os08g03640	1252	1033.21	622.20	1077.17	-0.73	1.19E-173	0.06	1.40E-02
RPP1B	LOC_Os08g02340	835	801.13	376.11	421.93	-1.09	2.14E-176	-0.93	2.57E-134
RPP0C	LOC_Os11g04070	1121	46.48	20.75	42.25	-1.16	8.12E-16	-0.14	3.18E-01
RPP2A	LOC_Os05g37330	819	1119.30	462.84	436.62	-1.27	0.00E+00	-1.36	0.00E+00
RPP2B	LOC_Os01g09510	886	758.95	287.02	301.03	-1.40	4.72E-263	-1.33	3.37E-243
RPP2D	LOC_Os02g32760	719	108.02	54.29	34.49	-0.99	4.14E-18	-1.65	3.52E-39
RPP2E	LOC_Os07g14750	0	0.00	0.00	0.71	0.00	0.00E+00	9.46	3.44E-01
RPSAb	LOC_Os07g42450	1348	204.11	132.86	172.82	-0.62	4.31E-28	-0.24	9.15E-06
RPSAa	LOC_Os03g08440	1445	220.85	162.31	333.17	-0.44	4.96E-18	0.59	1.30E-11
RPS2A	LOC_Os07g10660	1223	69.88	51.98	68.03	-0.43	2.87E-05	-0.04	7.08E-01
RPS2B	LOC_Os03g59310	1113	498.89	216.46	277.01	-1.20	9.93E-172	-0.85	1.24E-96
RPS3B	LOC_Os03g38000	1286	395.52	258.83	377.47	-0.61	2.97E-50	-0.07	1.04E-01
RPS3A	LOC_Os07g41750	1096	111.44	72.40	76.20	-0.62	5.72E-13	-0.55	8.81E-11
RPS3Aa	LOC_Os03g10340	1914	187.19	250.66	330.03	0.42	0.00E+00	0.82	0.00E+00
RPS4A	LOC_Os02g01560	1307	1864.81	723.90	886.78	-1.37	0.00E+00	-1.07	0.00E+00
RPS4B	LOC_Os01g25610	1242	599.80	230.92	366.76	-1.38	3.07E-283	-0.71	4.29E-95
11687.m02656	LOC_Os11g29190	1120	483.18	318.10	414.56	-0.60	4.31E-52	-0.22	6.60E-09
11667.m00007	LOC_Os01g01060	920	248.20	155.18	233.74	-0.68	2.35E-27	-0.09	1.70E-01
RPS6A	LOC_Os07g42950	753	773.55	378.77	612.15	-1.03	4.94E-140	-0.34	7.82E-20
RPS6B	LOC_Os03g27260	1101	578.19	331.26	566.60	-0.80	7.09E-101	-0.03	4.68E-01
RPS7A	LOC_Os03g18580	579	187.73	75.68	83.45	-1.31	6.87E-39	-1.17	9.56E-33
RPS7B	LOC_Os03g18570	968	420.02	208.39	247.60	-1.01	5.00E-95	-0.76	3.54E-59
RPS7C	LOC_Os05g27940	1132	303.75	166.52	224.17	-0.87	2.34E-62	-0.44	4.28E-19
RPS8A	LOC_Os04g28180	1054	1512.13	565.84	764.62	-1.42	0.00E+00	-0.98	0.00E+00
RPS10C	LOC_Os01g73160	540	330.36	98.26	77.92	-1.75	7.39E-97	-2.08	2.63E-122
11668.m03261	LOC_Os02g34460	894	87.06	31.55	35.44	-1.46	4.63E-33	-1.30	1.03E-27
11670.m03387	LOC_Os04g35090	552	628.63	348.69	385.50	-0.85	7.37E-61	-0.71	2.53E-44
RPS12C	LOC_Os07g12650	2018	115.46	39.56	51.38	-1.55	8.79E-106	-1.17	4.60E-69
RPS12A	LOC_Os07g05580	417	657.83	290.02	291.60	-1.18	1.19E-82	-1.17	9.15E-82
RPS13A	LOC_Os08g02410	952	677.75	341.60	417.81	-0.99	7.87E-145	-0.70	3.96E-80
RPS13A	LOC_Os08g02400	869	142.08	115.51	88.38	-0.30	3.71E-04	-0.68	1.55E-15
RPS13B	LOC_Os07g38540	910	51.24	44.30	38.68	-0.21	1.60E-01	-0.41	3.26E-03
RPS14B	LOC_Os02g06700	948	780.04	329.77	494.84	-1.24	1.04E-239	-0.66	2.04E-82
RPS14A	LOC_Os02g33140	980	366.44	161.94	167.02	-1.18	1.74E-107	-1.13	5.09E-101
RPS15D	LOC_Os03g58430	528	338.81	211.24	265.45	-0.68	8.66E-22	-0.35	2.30E-07
RPS15Aa	LOC_Os07g10720	789	976.14	408.34	563.34	-1.26	1.94E-254	-0.79	4.54E-119
RPS15Ae	LOC_Os02g15610	868	19.50	17.44	30.05	-0.16	5.72E-01	0.62	1.10E-03
RPS16A	LOC_Os11g03400	900	406.02	166.95	260.26	-1.28	7.16E-125	-0.64	1.45E-39
RPS16A	LOC_Os12g03090	1002	226.90	143.75	229.78	-0.66	6.34E-26	0.02	7.76E-01
RPS17A	LOC_Os10g27190	870	322.36	165.01	176.18	-0.97	5.11E-61	-0.87	1.71E-51
RPS17D	LOC_Os03g01900	721	38.67	43.54	73.23	0.17	3.81E-01	0.92	2.04E-11

RPS18A	LOC_Os03g58050	1017	712.10	364.74	532.22	-0.97	3.34E-156	-0.42	2.35E-36
RPS19C	LOC_Os03g31090	441	260.78	114.17	132.06	-1.19	1.74E-35	-0.98	3.25E-26
RPS20A	LOC_Os03g14530	753	171.68	68.98	65.66	-1.32	2.19E-46	-1.39	2.54E-50
RPS20C	LOC_Os10g08930	814	518.85	256.43	346.33	-1.02	1.40E-99	-0.58	1.43E-38
RPS20B	LOC_Os06g04290	774	261.58	128.66	186.25	-1.02	1.30E-48	-0.49	4.86E-14
RPS21C	LOC_Os03g22460	886	706.32	243.86	281.89	-1.53	6.93E-280	-1.33	4.38E-224
RPS23B	LOC_Os03g60400	429	368.65	169.36	233.47	-1.12	3.61E-44	-0.66	2.00E-18
RPS24B	LOC_Os02g13530	930	192.89	168.07	274.58	-0.20	4.37E-03	0.51	1.23E-11
11681.m03559	LOC_Os09g39540	716	140.69	125.29	111.73	-0.17	8.87E-02	-0.33	2.80E-04
RPS26B	LOC_Os01g60790	855	301.23	185.05	149.52	-0.70	8.69E-33	-1.01	1.95E-60
RPS26A	LOC_Os05g39960	959	644.25	304.88	321.33	-1.08	3.35E-160	-1.00	4.60E-142
11670.m02628	LOC_Os04g27860	261	211.73	119.04	186.36	-0.83	4.64E-10	-0.18	1.58E-01
RPS27A	LOC_Os04g32710	138	109.43	56.58	127.53	-0.95	4.12E-04	0.22	3.62E-01
RPS27Ab	LOC_Os01g22490	895	797.30	478.90	555.79	-0.74	5.48E-97	-0.52	9.89E-53
RPS27Ac	LOC_Os05g06770	859	443.56	383.78	508.12	-0.21	6.50E-06	0.20	4.22E-06
RPS28A	LOC_Os03g07100	958	58.38	19.96	10.69	-1.55	6.30E-26	-2.45	2.75E-47
RPS29A	LOC_Os11g41610	171	278.52	104.36	124.44	-1.42	1.75E-19	-1.16	1.15E-14
RPS30B	LOC_Os06g07580	583	662.66	295.99	368.84	-1.16	3.48E-113	-0.85	4.86E-67

Supplementary Table S4. Significant pathways in L1 plants detected by KEGG analysis.

#	Significant Pathways	DEGs with pathway annotation (3729)	All genes with pathway annotation (18735)	P value	Q value	Pathway ID
1	Phosphatidylinositol signaling system	58 (1.56%)	132 (0.7%)	2.82E-10	2.44E-08	ko04070
2	Endocytosis	65 (1.74%)	174 (0.93%)	6.52E-08	4.24E-06	ko04144
3	Cell cycle	86 (2.31%)	261 (1.39%)	4.11E-07	1.19E-05	ko04110
4	Aminoacyl-tRNA biosynthesis	39 (1.05%)	92 (0.49%)	6.96E-07	1.81E-05	ko00970
5	Oocyte meiosis	83 (2.23%)	260 (1.39%)	2.72E-06	5.94E-05	ko04114
6	Regulation of actin cytoskeleton	50 (1.34%)	135 (0.72%)	2.74E-06	5.94E-05	ko04810
7	Inositol phosphate metabolism	38 (1.02%)	97 (0.52%)	9.31E-06	1.61E-04	ko00562
8	Long-term potentiation	45 (1.21%)	125 (0.67%)	1.96E-05	2.83E-04	ko04720
9	Progesterone-mediated oocyte maturation	45 (1.21%)	127 (0.68%)	3.09E-05	4.02E-04	ko04914
10	Focal adhesion	35 (0.94%)	95 (0.51%)	9.21E-05	1.04E-03	ko04510
11	Non-homologous end-joining	10 (0.27%)	15 (0.08%)	1.08E-04	1.17E-03	ko03450
12	Bacterial secretion system	10 (0.27%)	16 (0.09%)	2.36E-04	2.45E-03	ko03070
13	Tight junction	32 (0.86%)	89 (0.48%)	3.02E-04	2.80E-03	ko04530
14	Nicotinate and nicotinamide metabolism	12 (0.32%)	22 (0.12%)	3.30E-04	2.86E-03	ko00760
15	MAPK signaling pathway	50 (1.34%)	160 (0.85%)	4.25E-04	3.57E-03	ko04010
16	Regulation of autophagy	33 (0.88%)	96 (0.51%)	6.24E-04	5.07E-03	ko04140
17	Nucleotide excision repair	54 (1.45%)	181 (0.97%)	8.90E-04	6.80E-03	ko03420
18	Ribosome	112 (3%)	436 (2.33%)	1.76E-03	1.14E-02	ko03010
19	Peroxisome	47 (1.26%)	159 (0.85%)	2.26E-03	1.44E-02	ko04146
20	Glycosylphosphatidylinositol(GPI)-anchor biosynthesis	11 (0.29%)	23 (0.12%)	2.39E-03	1.44E-02	ko00563
21	Phototransduction	17 (0.46%)	43 (0.23%)	2.39E-03	1.44E-02	ko04744
22	Photosynthesis - antenna proteins	9 (0.24%)	17 (0.09%)	2.48E-03	1.47E-02	ko00196
23	Chemokine signaling pathway	25 (0.67%)	73 (0.39%)	2.86E-03	1.64E-02	ko04062
24	Amino sugar and nucleotide sugar metabolism	63 (1.69%)	228 (1.22%)	2.90E-03	1.64E-02	ko00520
25	ErbB signaling pathway	20 (0.54%)	56 (0.3%)	4.22E-03	2.19E-02	ko04012
26	mTOR signaling pathway	31 (0.83%)	99 (0.53%)	4.68E-03	2.30E-02	ko04150
27	Circadian rhythm - plant	54 (1.45%)	194 (1.04%)	4.69E-03	2.30E-02	ko04712
28	ABC transporters	37 (0.99%)	124 (0.66%)	5.28E-03	2.54E-02	ko02010
29	Jak-STAT signaling pathway	9 (0.24%)	19 (0.1%)	6.41E-03	3.03E-02	ko04630
30	Dorso-ventral axis formation	15 (0.4%)	40 (0.21%)	7.50E-03	3.42E-02	ko04320
31	Calcium signaling pathway	48 (1.29%)	173 (0.92%)	7.83E-03	3.49E-02	ko04020
32	Purine metabolism	61 (1.64%)	229 (1.22%)	7.92E-03	3.49E-02	ko00230
33	Gap junction	23 (0.62%)	73 (0.39%)	1.26E-02	4.91E-02	ko04540
34	Adherens junction	23 (0.62%)	73 (0.39%)	1.26E-02	4.91E-02	ko04520
35	Protein processing in endoplasmic reticulum	112 (3%)	463 (2.47%)	1.26E-02	4.91E-02	ko04141

* We use "FDR ≤ 0.001 and the absolute value of log2Ratio ≥ 1" as the threshold to judge the significance of gene expression difference

Supplementary Table S5. Significant pathways in L2 plants detected by KEGG analysis.

#	Significant Pathways	DEGs with pathway annotation (6131)	All genes with pathway annotation (18735)	P value	Q value	Pathway ID
1	Aminoacyl-tRNA biosynthesis	55 (0.9%)	92 (0.49%)	8.57E-08	7.80E-06	ko00970
2	Regulation of actin cytoskeleton	73 (1.19%)	135 (0.72%)	2.36E-07	1.61E-05	ko04810
3	Lysine degradation	31 (0.51%)	49 (0.26%)	1.12E-05	5.08E-04	ko00310
4	Non-homologous end-joining	13 (0.21%)	15 (0.08%)	2.50E-05	8.52E-04	ko03450
5	Photosynthesis - antenna proteins	14 (0.23%)	17 (0.09%)	3.66E-05	1.11E-03	ko00196
6	Focal adhesion	50 (0.82%)	95 (0.51%)	4.64E-05	1.27E-03	ko04510
7	Phosphatidylinositol signaling system	65 (1.06%)	132 (0.7%)	5.80E-05	1.44E-03	ko04070
8	Purine metabolism	103 (1.68%)	229 (1.22%)	6.75E-05	1.44E-03	ko00230
9	Cell cycle	115 (1.88%)	261 (1.39%)	7.77E-05	1.52E-03	ko04110
10	Fatty acid metabolism	38 (0.62%)	69 (0.37%)	1.05E-04	1.68E-03	ko00071
11	Nicotinate and nicotinamide metabolism	16 (0.26%)	22 (0.12%)	1.42E-04	2.02E-03	ko00760
12	Adherens junction	39 (0.64%)	73 (0.39%)	2.03E-04	2.64E-03	ko04520
13	Peroxisome	73 (1.19%)	159 (0.85%)	3.46E-04	4.10E-03	ko04146
14	Tight junction	45 (0.73%)	89 (0.48%)	3.60E-04	4.10E-03	ko04530
15	Oocyte meiosis	110 (1.79%)	260 (1.39%)	7.08E-04	7.16E-03	ko04114
16	Pyruvate metabolism	48 (0.78%)	101 (0.54%)	1.37E-03	1.16E-02	ko00620
17	Endocytosis	75 (1.22%)	174 (0.93%)	2.58E-03	1.95E-02	ko04144
18	Nucleotide excision repair	77 (1.26%)	181 (0.97%)	3.46E-03	2.46E-02	ko03420
19	ABC transporters	55 (0.9%)	124 (0.66%)	4.39E-03	2.94E-02	ko02010
20	Glyoxylate and dicarboxylate metabolism	29 (0.47%)	58 (0.31%)	4.68E-03	2.99E-02	ko00630
21	Progesterone-mediated oocyte maturation	56 (0.91%)	127 (0.68%)	4.74E-03	2.99E-02	ko04914
22	Gap junction	35 (0.57%)	73 (0.39%)	4.82E-03	2.99E-02	ko04540
23	Pathogenic Escherichia coli infection	43 (0.7%)	94 (0.5%)	5.67E-03	3.37E-02	ko05130
24	Chloroalkane and chloroalkene degradation	19 (0.31%)	35 (0.19%)	6.81E-03	3.72E-02	ko00625
25	Other glycan degradation	20 (0.33%)	38 (0.2%)	8.69E-03	4.50E-02	ko00511
26	Regulation of autophagy	43 (0.7%)	96 (0.51%)	8.89E-03	4.50E-02	ko04140
27	Glutathione metabolism	63 (1.03%)	149 (0.8%)	8.91E-03	4.50E-02	ko00480
28	Propanoate metabolism	24 (0.39%)	48 (0.26%)	9.63E-03	4.75E-02	ko00640
29	Ascorbate and aldarate metabolism	47 (0.77%)	107 (0.57%)	9.91E-03	4.75E-02	ko00053

* We use "FDR ≤ 0.001 and the absolute value of log2Ratio ≥ 1" as the threshold to judge the significance of gene expression difference
